# Spacer-free BODIPY fluorogens in antimicrobial peptides for direct imaging of fungal infection in human tissue

**DOI:** 10.1038/ncomms10940

**Published:** 2016-03-09

**Authors:** Lorena Mendive-Tapia, Can Zhao, Ahsan R. Akram, Sara Preciado, Fernando Albericio, Martin Lee, Alan Serrels, Nicola Kielland, Nick D Read, Rodolfo Lavilla, Marc Vendrell

**Affiliations:** 1 Institute for Research in Biomedicine, Barcelona Science Park, Baldiri Reixac 10-12, Barcelona 08028, Spain; 2 Manchester Fungal Infection Group, Institute of Inflammation and Repair, University of Manchester, CTF Building, Grafton St, Manchester M13 9NT, UK; 3 MRC/UoE Centre for Inflammation Research, University of Edinburgh, 47 Little France Crescent, Edinburgh EH16 4TJ, UK; 4 Department Organic Chemistry, University of Barcelona, Martí i Franqués 1-11, Barcelona 08028, Spain; 5 CIBER-BBN, Networking Centre for Bioengineering, Biomaterials and Nanomedicine, Baldiri Reixac 10-12, Barcelona 08028, Spain; 6 School of Chemistry, University of KwaZulu-Natal, Durban 4001, South Africa; 7 Edinburgh Cancer Research Centre, University of Edinburgh, Crewe South Road, Edinburgh EH4 2XR, UK; 8 Laboratory of Organic Chemistry, Faculty of Pharmacy, University of Barcelona, Barcelona Science Park, Baldiri Reixac 10-12, Barcelona 08028, Spain

## Abstract

Fluorescent antimicrobial peptides are promising structures for *in situ*, real-time imaging of fungal infection. Here we report a fluorogenic probe to image *Aspergillus fumigatus* directly in human pulmonary tissue. We have developed a fluorogenic Trp-BODIPY amino acid with a spacer-free C-C linkage between Trp and a BODIPY fluorogen, which shows remarkable fluorescence enhancement in hydrophobic microenvironments. The incorporation of our fluorogenic amino acid in short antimicrobial peptides does not impair their selectivity for fungal cells, and enables rapid and direct fungal imaging without any washing steps. We have optimized the stability of our probes in human samples to perform multi-photon imaging of *A. fumigatus* in *ex vivo* human tissue. The incorporation of our unique BODIPY fluorogen in biologically relevant peptides will accelerate the development of novel imaging probes with high sensitivity and specificity.

## Abstract (web summary)

Functionalizing antimicrobial peptides with fluorescent groups is a useful strategy for imaging infection, but the tag can alter the performance of the probe. Here, the authors report a spacer-free method to directly functionalise an amino acid with a fluorogenic group and prepare peptide-based imaging agents for fungal infection.

## Graphical abstract

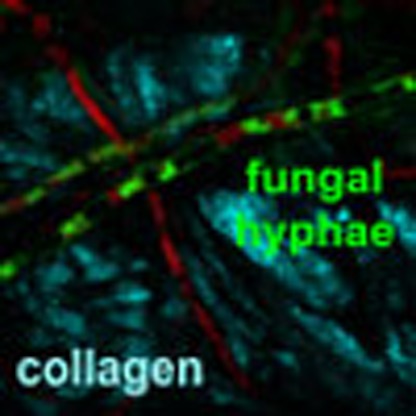

Invasive pulmonary aspergillosis (IPA) is a highly fatal disease in immunocompromised patients. IPA results from the infection with the fungal pathogen *Aspergillus fumigatus*, and it is a frequent cause of fungal pneumonia with mortality rates up to 40% (ref. 1). Current diagnostic approaches for IPA rely on histological analysis, cultures from bronchoalveolar lavage fluid and sampling peripheral blood^2^. These methods are fraught with problems of upper airway contamination and diagnostic delays, by which time the disease may have progressed or been treated empirically with inappropriate drugs. Moreover, blood markers are unlikely to provide useful information about events deep in pulmonary tissue, especially in patients with multi-system disease, such as immunosuppressed patients affected by IPA. These limitations of current diagnostic tools have prompted the development of imaging probes that can provide *in situ* and real-time information on the progression of infection^3^,^4^,^5^,^6^. Fluorescent probes based on antibiotics and antimicrobial peptides are chemical entities with enormous potential for imaging infection sites due to their high selectivity for microbial cell structures over mammalian cells^7^,^8^,^9^,^10^,^11^. van Oosten *et al.*^12^ recently reported a near-infrared fluorescently labelled vancomycin for real-time *in vivo* imaging of bacterial infections in a mouse myocitis model. Similarly, Thiberville and co-workers have described fluorescein-conjugated peptides to visualize fungal biofilms in immunosupressed rats using fibre-based microendoscopy^13^. These probes have been prepared by conjugating peptides of interest to suitable fluorophores via chemical spacers. While such approaches have been useful to functionalise long peptides or proteins^14^, alternative strategies are needed for shorter peptides, where relevant modifications can compromise their specificity. Our group and others have studied the mechanism of action of Peptide AntiFungal 26 (PAF26), a synthetic antimicrobial hexapeptide with high affinity for fungal cells and selectivity over bacterial and mammalian cells^15^,^16^. We envisaged that fluorescent analogues of PAF26 would enable imaging of fungal infection sites provided that the main recognition features of PAF26 remained unaffected after labelling. However, the incorporation of fluorophores in short antimicrobial peptides is challenging as chemical modifications are likely to alter the distribution of positive charges as well as their amphipathic character. PAF26 has a highly conserved sequence with a *C*-terminal hydrophobic domain (Trp–Phe–Trp) and an *N*-terminal cationic domain (Arg–Lys–Lys) that are essential to exert its antifungal action. Site-specific peptide labelling can be achieved by incorporation of amino acids with bio-orthogonal^17^,^18^,^19^ or fluorogenic groups^21^,^22^,^23^. Fluorogenic amino acids are advantageous in that they provide high signal-to-noise ratios without the need for washing or additional labelling steps. A number of fluorogenic amino acids have been reported^24^,^25^,^26^, but most exhibit inherent limitations as fluorophores (for example, short emission wavelengths, low extinction coefficients and compromised cell permeability). We have developed a spacer-free fluorogenic amino acid based on the 4,4-difluoro-4-bora-3*a*,4*a*-diaza-*s*-indacene (BODIPY) scaffold, and incorporated it in the hydrophobic domain of PAF26 to maintain the recognition features of the peptide while providing an excellent reporter of the interaction with fungal cells. This innovative approach has rendered fluorogenic BODIPY-labelled antimicrobial peptides as highly stable probes to image *A. fumigatus* directly in *ex vivo* human tissue.

## Results

### Design and synthesis of a Trp-BODIPY fluorogenic amino acid

**Figure 1:**
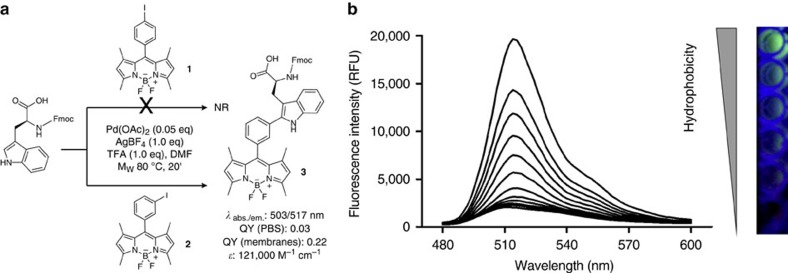
A Trp-BODIPY fluorogenic amino acid. (**a**) Synthetic scheme and spectral properties of the Trp-BODIPY fluorogenic amino acid **3** (NR: no reaction). (**b**) The amino acid **3** displays strong fluorogenic behaviour in phospholipid membranes. Spectra of compound **3** (10 μM) were recorded after incubation with PC:cholesterol (7:1) liposome suspensions in PBS ranging from 3.75 to 0.004 mg ml^−1^ of PC in two-fold serial dilutions, *λ*_exc._: 450 nm. PBS alone was used as a negative control for a non-hydrophobic environment. On the right-hand side, pictures of the fluorescence emission of **3** under excitation with a 365 nm UV-lamp in PC:cholesterol liposome suspensions with increasing PC content (from top to bottom: 3.75, 1.88, 0.94, 0.47, 0.23 and 0 (only PBS) mg ml^−1^ of PC).

BODIPY is a fluorescent structure with excellent cell permeability and photophysical properties^27^,^28^. Moreover, the BODIPY scaffold can be derivatized with radioisotopes to prepare multimodal agents for both optical imaging and positron emission tomography^29^,^30^, enabling quantitative whole-body imaging with high sensitivity^31^,^32^. Multimodal agents, which are designed to be compatible with complementary imaging modalities, are excellent tools to achieve good spatial resolution and specificity without compromising high sensitivity^33^. Despite the numerous BODIPY derivatives described to date^34^,^35^,^36^,^37^,^38^, there are no reports of BODIPY-based fluorogenic amino acids. Environmentally sensitive fluorogens can be prepared by direct conjugation of the BODIPY core to electron-rich groups leading to photo-induced electron transfer quenching^39^,^40^,^41^. We envisaged that the direct coupling of the indole group of Trp to the BODIPY core would render a fluorogenic amino acid with potential to replace Trp in the preparation of fluorogenic antimicrobial peptides. Our group has recently described some Pd-catalysed C-H activation^42^,^43^,^44^ as an efficient way to arylate the indole C_2_ position^45^ of Trp and prepare Trp-derivatized peptides and peptidomimetics^46^,^47^,^48^. In this way, we synthesized two BODIPY iodide derivatives (**1** and **2**, Fig. 1a) in good yields using our recently developed procedures and assessed their reactivity in Pd-catalysed C_2_-arylation of Fmoc-Trp-OH. Notably, only the conjugate **3** was obtained from the *m*-iodophenyl-BODIPY (**2**)^49^, while the corresponding *p*-iodophenyl **1** was unreactive, reflecting electronic preferences (Fig. 1a and Supplementary Discussion). We further optimized the gram-scale synthesis of **3** using microwave-assisted irradiation to readily isolate the fluorogenic amino acid as a solid stable compound with 74% yield, suitably protected to be directly used in solid-phase peptide synthesis (SPPS).

### Synthesis and evaluation of fluorogenic antifungal peptides

**Figure 2:**
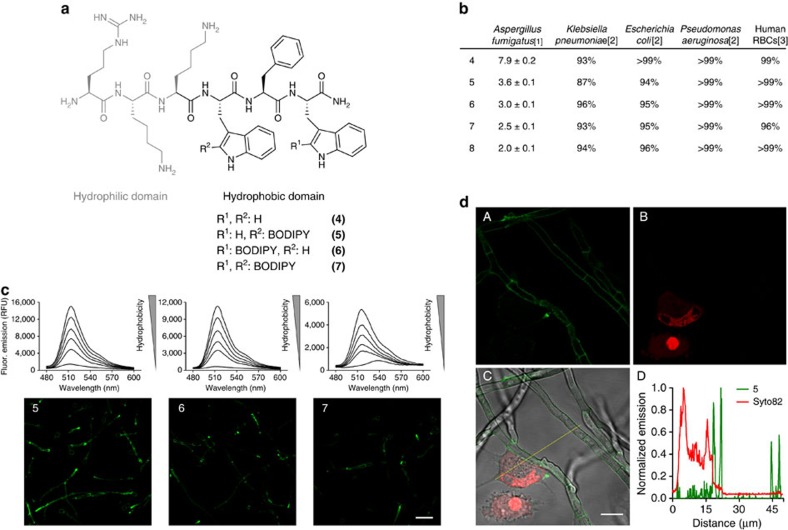
Fluorogenic peptides for live cell imaging of *A.fumigatus* in co-culture with human lung epithelial cells. (**a**) Chemical structures of non-labelled and fluorogenic linear peptides (**4**-**7**), highlighting the two conserved hydrophilic (grey) and hydrophobic (black) domains of Peptide Antifungal 26 (PAF26). (**b**) Activity of antimicrobial peptides in *A. fumigatus*, several bacterial strains and in human RBCs.[1] IC_50_ (μM) values represented as means±s.e.m. from *n*=3, [2] cell viability upon 16 h incubation with **4**–**8** at their respective IC_50_ concentrations (*n*=3), [3] cell viability upon 1 h incubation with **4**–**8** at their respective IC_50_ concentrations (*n*=3). (**c**) Fluorogenic behaviour of **5**–**7** (10 μM) in phosphatidylcoline (PC):cholesterol (7:1) liposome suspensions in PBS ranging from 3.75 to 0.004 mg ml^−1^ of PC in two-fold serial dilutions (*λ*_exc._: 450 nm), and wash-free live cell images of *A. fumigatus* at 37 °C using fluorescence confocal microscopy after incubation with peptides **5**–**7** (5 μM). Scale bar, 20 μm. (**d**) Peptide **5** (5 μM, green) and Syto82 (2.5 μM, red counterstain for lung epithelial cells) were incubated in co-cultures of *A. fumigatus* and human lung A549 epithelial cells and imaged under a fluorescence confocal microscope at 37 °C without any washing steps. Fluorescence staining of **5** (A), Syto82 (B), merged (C) and plot profile analysis (D) of peptide **5** (green) and Syto82 (red) from image C. Scale bar, 10 μm.

The amino acid **3** displayed characteristic absorption and emission wavelengths of BODIPY probes as well as very high extinction coefficients (Fig. 1a, Supplementary Figs 1,2). Next we evaluated the properties of **3** as a fluorogenic probe and its potential to report interactions of antimicrobial peptides with fungal cells. Many antimicrobial peptides, including PAF26, recognize molecular components of the microbial cell membrane and accumulate in lipophilic intracellular compartments. Therefore, we examined the fluorescence spectra of **3** in phospholipid bilayer membranes that mimic such microenvironments. As shown in Fig. 1b, the BODIPY core embedded in **3** displayed remarkable fluorogenic behaviour with strong fluorescence emission upon binding to phospholipid membranes. In view of the properties of **3** as a fluorogenic surrogate of Trp, we prepared fluorogenic derivatives of PAF26 by SPPS. Since the sequence of PAF26 (**4**, Fig. 2a) contains two Trp residues, we synthesized all three possible combinations (**5**–**7**, Fig. 2a) to assess the impact of the amino acid **3** at different positions of the antimicrobial peptide. The amino acid **3** proved to be fully compatible with SPPS as it tolerates standard Fmoc deprotection and coupling conditions as well as mildly acidic (that is, 1% trifluoroacetic acid) cleavage cocktails for acid-labile solid supports (for example, Sieber amide and chlorotrityl-based polystyrene resins) without observing any degradation (Supplementary Methods and Supplementary Fig. 20). Being mildly acidic conditions harmless to the BODIPY core^50^, peptides **5**–**7** were prepared using conventional SPPS protocols in a Sieber amide polystyrene resin. Molecular simulation models of both labelled and non-labelled peptides corroborated that the introduction of BODIPY scaffolds in the hydrophobic domain of PAF26 did not disrupt the conformation and hydrogen bonding pattern of the original peptide (Supplementary Fig. 3). Next we determined the activity of the peptides **4**–**7** in *A. fumigatus* as well as in bacterial strains and human RBCs as an indication of their affinity for both microbial and human cells. We included *Klebsiella pneumoniae*, *Escherichia coli* and *Pseudomonas aeruginosa* as clinically relevant bacterial strains commonly found in hospitalized pulmonary infections^51^. Likewise, we tested the activity of **4**–**7** in human RBCs, because positively charged peptides are potential haemolytic agents^52^. Remarkably, the incorporation of **3** in the hydrophobic domain of PAF26 rendered peptides (**5**–**7**) with slightly higher affinity for *A. fumigatus* than the non-labelled PAF26 peptide (**4**) (Fig. 2b and Supplementary Fig. 4). The marginal activity of PAF26 in bacterial and human cells was also maintained in all fluorogenic analogues (Fig. 2b, Supplementary Figs 5,6). Altogether, these results validate the direct C-C conjugation of BODIPY fluorogens to the C_2_ position of the indole ring of Trp as a novel labelling approach with minimal interference in the molecular recognition properties of PAF26 while providing a suitable tag to report the interaction with *A. fumigatus*.

### Imaging *Aspergillus fumigatus* in co-culture with human cells

Peptides **5**–**7** exhibited similar spectral properties to **3** with an equally strong fluorogenic behaviour in phospholipid membranes (Fig. 2c and Supplementary Fig. 7). Double-labelled peptide **7** displayed a weaker fluorescence response than mono-labelled peptides (**5**, **6**), partially due to the self-quenching derived from two neighbouring BODIPY fluorophores. In view of the excellent properties of **5**-**7** as fungi-targeting fluorogenic peptides, we evaluated them as live cell imaging agents of *A. fumigatus*. Peptides **5** and **6** brightly stained fungal cells, whereas **7** showed significantly weaker fluorescence, in accordance with its lower fluorogenicity (Fig. 2c). As negative controls, we assessed the activity and imaging properties of fluorogenic derivatives of PAF26 replacing some of the key residues for their interaction with fungal cells^53^. Peptide **5a**, which lacks the hydrophilic domain of PAF26, showed poor activity and staining in *A. fumigatus* (Supplementary Fig. 8). Similar results were obtained when we examined the activity and staining properties of the single BODIPY amino acid **3** (Supplementary Fig. 8). We also synthesized peptide **5b**, including less non-polar residues in the hydrophobic domain, which exhibited reduced activity and brightness in *A. fumigatus* (Supplementary Fig. 8). These observations confirmed the importance of embedding the amino acid **3** within the full amphipathic sequence of PAF26 in order to efficiently interact with the cell membrane of *A. fumigatus*.

We further used peptide **5** to image live *A. fumigatus* in co-cultures with human lung epithelial cells. As shown in Fig. 2d, the fluorogenic properties of **5** enabled direct live fungal cell imaging without the need of any washing steps. Furthermore, we counterstained lung epithelial cells with the red fluorescent dye Syto82 and performed plot profile analysis to confirm that **5** specifically labelled *A. fumigatus* without staining human lung epithelial cells (Fig. 2d).

### Probe optimization for direct *ex vivo* tissue imaging

**Figure 3:**
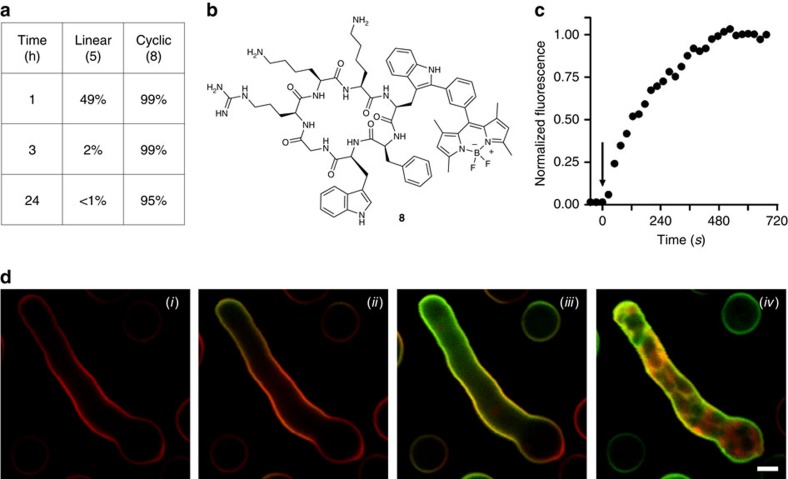
The cyclic peptide 8 is a highly stable fluorogenic agent for high-resolution imaging of *A. fumigatus*. (**a**) Comparative chemical stability of mono-labelled BODIPY linear (**5**) and cyclic (**8**) PAF26 analogues in human bronchoalveolar lavage samples from patients with acute respiratory distress syndrome. (**b**) Chemical structure of the cyclic BODIPY-labelled peptide **8**. (**c**) Kinetic analysis (from time-lapse imaging in **d** of the fluorescence signal of compound **8** (2 μM) in the cell membrane of *A. fumigatus* (arrow points at the addition time for compound **8**). (**d**) Time-lapse high-resolution imaging of *A. fumigatus* upon incubation with a cell membrane counterstain (red) and compound **8** (2 μM, green) for 0 min (*i*), 1 min (*ii*), 3 min (*iii*) and 10 min (*iv*) (see Supplementary Movie 3). Scale bar, 2.5 μm.

Direct tissue imaging of infection sites is often hampered by the high concentration of proteolytic enzymes^54^, which can compromise the integrity of imaging agents. Hence, we decided to examine the chemical stability of peptide **5** in human bronchoalveolar lavages from patients with acute respiratory dystress syndrome to assess the potential for *ex vivo* human tissue imaging. The linear peptide **5** was rapidly degraded in human lavages with a half-life shorter than 60 min (Fig. 3a, Supplementary Figs 9,10). To enhance the stability required for direct *ex vivo* imaging in human pulmonary tissue, we synthesized **8** as the corresponding BODIPY-labelled cyclic analogue (Fig. 3b). Cyclic peptides do not contain free *N*- and *C*-terminal groups, leading to increased resistance to degradation by proteases^55^,^56^. We synthesized compound **8** using 2-chlorotrityl polystyrene resin, which enabled the preparation and subsequent cleavage of the protected linear peptide under mild acidic conditions (Supplementary Fig. 11). Head-to-tail cyclization was performed in solution with 87% yield using HATU as the coupling reagent. We optimized the reaction conditions to remove all the protecting groups without affecting the BODIPY scaffold. Reduction of the protected peptide in H_2_ atmosphere with Pd(OH)_2_/C using mild acidic conditions led to the desired product with yields around 60% and purities over 90%. The peptide **8** showed around two-fold enhanced affinity for fungal cells compared with peptide **5**, and maintained very high selectivity over bacteria and human cells (Fig. 2b). A similar activity profile was observed for peptide **9**, the non-labelled analogue of peptide **8** (Supplementary Fig. 8). Peptide **9** showed slightly enhanced affinity for *A. fumigatus* when compared with the linear PAF26 sequence (**4**), and maintained high selectivity over bacteria and human RBCs (Supplementary Fig. 8). These observations are in line with the fact that peptide cyclization can restrict conformational flexibity, which often leads to enhanced affinity and activity^57^. Preliminar NMR analysis of **8** showed no evidence of relevant structural modifications with respect to the non-labelled peptide **9**, in agreement with molecular simulations (Supplementary Fig. 3). Importantly, the peptide **8** remained intact after 24 h in human bronchoalveolar lavages from patients with acute respiratory dystress syndrome (Fig. 3a, Supplementary Figs 9,10). The peptide **8** also displayed stronger fluorogenic response than the linear peptides (**5**,**6**) and remarkable fluorescence emission in phospholipid membranes with quantum yields reaching 30% (Supplementary Figs 12,13). In addition to *A. fumigatus*, we examined the ability of peptide **8** to stain different fungal strains (Supplementary Fig. 14). While we observed slight differences in fluorescence intensity between strains, peptide **8** stained most fungal cells, indicating its potential as a probe for imaging fungal infection sites of variable origin. We also employed **8** to image *A. fumigatus* that had been pre-treated or not with an excess of non-labelled PAF26 (**4**) (Supplementary Fig. 15 and Supplementary Movies 1,2). Cells that were pre-treated with compound **4** showed significantly lower staining when exposed to the same concentration of peptide **8**, confirming the specificity of our fluorogenic cyclic structure. We also confimed that the peptide **8** brightly stained *A. fumigatus* in co-cultures with human lung epithelial cells (Supplementary Fig. 16). All these observations assert the cyclic peptide **8** as a fluorogenic probe with high stability in lavage samples from patients with multi-system respiratory disease and potential for direct *ex vivo* imaging of *A. fumigatus* in human pulmonary tissue.

### *Ex vivo* imaging of *Aspergillus fumigatus* in human tissue

**Figure 4:**
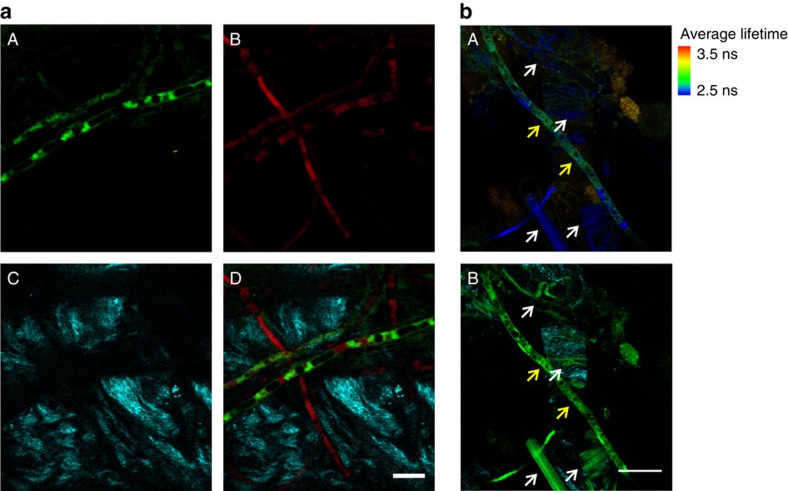
Multi-photon fluorescence microscopy of *ex vivo* human pulmonary tissue after incubation with RFP-expressing *A. fumigatus*. (**a**) Multi-photon microscope images from peptide **8** (5 μM) (A), RFP-expressing *A. fumigatus* (B), second harmonic generation from collagen fibres (C) and merged (D) in *ex vivo* human lung tissue. Scale bar, 10 μm. (**b**) (A) Fluorescence lifetime image of **8**-stained *A. fumigatus* in *ex vivo* human lung tissue. White arrows point autofluorescent tissue structures and yellow arrows point **8**-stained fungal cells. (B) Corresponding fluorescence image of **8**-stained *A. fumigatus* (green) and collagen fibres (second harmonic generation, cyan) for the fluorescence lifetime image in A. Scale bar, 20 μm.

Next we employed the peptide **8** for high-resolution imaging of *A. fumigatus*. Time-lapse imaging showed the fluorogenic response of **8** upon interaction with the fungal cell membrane and after being internalized and accumulated in lipid-rich intracellular compartments (Fig. 3d and Supplementary Movie 3). The kinetic analysis shows that the peptide **8** labelled fungal cells very rapidly, within few minutes after addition of the probe and without requiring any washing steps (Fig. 3c). Moreover, the peptide **8** showed no cytotoxicity in lung epithelial cells, even at high concentrations (Supplementary Fig. 17). In view of these properties, we employed the peptide **8** for direct imaging of *A. fumigatus* in human pulmonary tissue using multi-photon microscopy. In order to confirm the specific staning of **8**, we employed a transgenic strain of *A. fumigatus* expressing red fluorescent protein (RFP) in the cytoplasm. As shown in Fig. 4a, the peptide **8** (green) clearly stained RFP-expressing *A. fumigatus* (red), which confirmed the selectivity of our probe. Multi-photon excitation enabled the acquisition of second harmonic generation (cyan) from the collagen structures of the fibrilar network of human pulmonary tissue. Furthermore, the examination of these samples by fluorescence lifetime imaging revealed that autofluorescent human tissue structures (for example, collagen and elastin)^58^, which could potentially overlap with the emission of BODIPY fluorogens, are readily distinguished from **8**-stained *A. fumigatus* by their fluorescence lifetimes (Fig. 4b). Altogether, these results validate our fluorogenic BODIPY-labelled cyclic peptide **8** as a highly stable imaging agent for direct and straightforward visualization of *A. fumigatus* in human tissue.

## Discussion

Peptides are excellent scaffolds for the development of imaging agents due to the highly specific molecular interactions with their respective targets. Since most peptides do not contain chemical groups that enable their direct visualization, they often need to be modified with reporters (for example, fluorophores) or reactive groups (for example, aldehydes, azides, alkynes and tetrazines) for further derivatization^59^. Unnatural amino acids containing bio-orthogonal tags can be incorporated at specific sites of peptide sequences by SPPS^60^. Likewise, the incorporation of genetically encoded unnatural amino acids in response to nonsense or frameshift codons has opened the possibility to synthesize protein and peptide structures with reactive groups for subsequent modification^61^. Bio-orthogonal approaches typically involve two-step labelling processes including a conjugation reaction (for example, ‘click' chemistry) followed by the removal of excess labelling agent. Recent advances in bio-orthogonal chemistry have led to fluorogenic labelling agents that emit a signal only after conjugation, thus reducing background fluorescence and washing steps^62^,^63^. Alternatively, and most commonly, peptides are derivatized by incorporation of fluorophores into their sequence so they can be directly used for imaging. Since fluorophores are typically bulkier structures, it is imperative that they are introduced at specific positions of the sequence without impairing the molecular recognition properties of the peptide. Many conjugation methods to attach fluorophores to peptides involve a chemical spacer and rely on the reactivity of polar groups (that is, amines, carboxylic acids, thiols, alcohols); however, these modifications often disrupt the hydrogen bonding pattern of the original peptide, having a detrimental effect on its biological properties.

In the present work, we have engineered a methodology to prepare fluorogenic peptides that relies on a unique Trp-BODIPY derivative (**3**, Fig. 1), which mimics the molecular interactions of the native Trp. The incorporation of a BODIPY group into the C_2_ position of Trp via a spacer-free C-C linkage does not affect the conformation and molecular interactions of the native amino acid, and introduces a fluorogenic tag that emits only in hydrophobic environments (Fig. 1). To assess the compatibility of our approach with SPPS and validate its utility to prepare peptide-based agents for imaging of fungal infection, we derivatized the antimicrobial hexapeptide PAF26, which shows high affinity for the membrane of fungal cells. PAF26 is an amphipathic peptide with highly conserved *C*-terminal hydrophobic and *N*-terminal cationic domains that are essential to exert its antifungal action^16^. Therefore, the derivatization of PAF26 is not straightforward since conventional labelling might alter the distribution of positive charges or its amphipathic character, resulting in a loss of activity and selectivity.

Analogues of PAF26 incorporating the fluorogenic amino acid **3** at specific sites in their sequence were prepared by SPPS and showed no impairment of the affinity and selectivity of the original peptide for fungal cells (Fig. 2). Our fluorogenic peptides were used for real-time imaging of several fungal pathogens, namely *Fusarium oxysporum*, *Candida albicans*, *Cryptococcus neoformans* and *A. fumigatus*, suggesting a potential common target for different fungal strains (Supplementary Fig. 14). Given that *A. fumigatus* is the fungal pathogen responsible for IPA, a highly fatal disease in immunocompromised patients, we focused our imaging studies in this fungal strain.

Notably, the minimal fluorescence background in aqueous media and strong fluorogenic behaviour of our probes enabled their use for direct and wash-free imaging of *A. fumigatus* (Fig. 2). Competition experiments with the corresponding non-labelled analogues and comparative studies with non-antifungal negative controls—lacking key residues for the interaction at fungal cells—confirmed the specificity of our PAF26-derived fluorogenic peptides (Supplementary Figs 8,15).

A major advantage of our methodology is its wide applicability to bioconjugation and peptide chemistry. The fluorogenic amino acid **3** and its peptide derivatives are compatible with most Fmoc-based SPPS protocols as they tolerate standard deprotection and coupling conditions as well as mildly acidic (that is, 1% trifluoroacetic acid) cleavage cocktails without observing any degradation. Whereas the precise impact of the amino acid **3** in the molecular recognition properties of labelled sequences needs to be examined on a case-by-case basis, we observed similar activities for labelled and non-labelled peptides in a relatively broad range of short antimicrobial sequences, which confirms the ability of the Trp-BODIPY amino acid **3** to behave as a Trp surrogate (Fig. 2 and Supplementary Fig. 8).

With these peptides being promising imaging agents for *in situ* detection of fungal pathogens in clinically relevant samples, we optimized their chemical stability to image *A. fumigatus* in *ex vivo* human pulmonary tissue. Our optimization studies yielded peptide **8** as a highly fluorogenic cyclic structure with bright fluorescence emission in fungal cells and excellent chemical integrity in samples with high proteolytic activity (Fig. 3 and Supplementary Figs 9,16). The excellent properties of **8** enabled its use in multi-photon and lifetime imaging for the direct visualization of *A. fumigatus* in *ex vivo* human tissue and its discrimination from autofluorescent tissue structures (Fig. 4).

Given that the fluorogenic amino acid **3** can be readily incorporated and has general applicability to both linear and cyclic peptides, we envisage that the introduction of our spacer-free BODIPY fluorogen in relevant peptides will become a transformative methodology to develop peptide-based imaging probes with high sensitivity and specificity. Furthermore, the extension of our methodology to other aromatic amino acids will create numerous opportunities for minimally invasive peptide tagging using synthetically available building blocks.

## Methods

### Chemical synthesis and characterization

Synthetic procedures and chemical characterization (NMR and high-performance liquid chromatography analysis) for all the probes are included in the Supplementary Information (Supplementary Figs 18–25).

### *In vitro* spectral measurements

Spectroscopic and quantum yield data were recorded on a Synergy HT spectrophotometer (Biotek). Compounds were dissolved at the indicated concentrations and spectra were recorded at room temperature. Spectra are represented as means from at least two independent experiments with *n*=3. Quantum yields were calculated by measuring the integrated emission area of the fluorescence spectra and comparing it to the area measured for fluorescein in basic ethanol as reference (QY: 0.97). Phosphatidylcoline (PC)-based liposome suspensions were purchased from Clodronateliposomes (Netherlands) and were prepared as previously reported^64^.

### IC_50_ determination in *Aspergillus fumigatus*

The *A. fumigatus* (strain CEA10, source: FGSC A1163) was grown on Vogel's medium at 37 °C for 5 days before the spores (conidia) were harvested. Peptides **4**–**8** were incubated at different concentrations with *A. fumigatus* conidia to reach a final volume of 100 μl per well. The final conidia concentration was 5 × 10^5^ cells ml^−1^ in 10% Vogel's medium. After 24 h incubation at 37 °C in 96 well-plates, fungal growth was determined by measuring OD_610nm_ in a spectrophotometer. IC_50_ values were determined using four parameter logistic regression. Data is represented as means±s.e.m from at least three independent experiments with *n*=3.

### Cell culture of fungal strains

*Neurospora crassa* (strain *74-OR23-1V*A, source: FGSC 2489) was grown on standard Vogel's agar (Vogel, 1956) at 25 °C under constant artificial light for 5 days. Conidia were collected using sterile dH_2_O and then diluted in 20% Vogel's liquid medium for imaging. *F. oxysporum* (strain *4287*, source: FGSC 9935) was grown in liquid potato dextrose broth (PDB) at 28 °C with shaking. Conidia were re-suspended in 20% Vogel's liquid medium and imaged after incubation for 12 h at 30 °C. *Can. albicans* (strain SC5314, source: ATCC MYA-2876) was grown on yeast peptone dextrose liquid medium at 30 °C for 12 h with shaking and then diluted using minimal medium (0.7% yeast nitrogen base plus 2% glucose) before imaging. *Cry. neoformans* (strain H99, source: FGSC 9487) was grown on yeast peptone dextrose agar at 30 °C for 3 days. To collect the cells for imaging, a single colony 1-2 mm in diameter was re-suspended in PBS and washed once with fresh PBS before imaging.

### *In vitro* measurements of antimicrobial activity

*P. aeruginosa* (ATCC 47085), *K. pneumoniae* (ATCC BAA1706) and *E. coli* (ATCC 25922) were grown on Lysogeny Broth (LB) agar plates and stored at 4 °C. For assays, a single colony of bacteria was taken into 10 mL liquid broth and incubated at 37 °C for 16 h. Cultures were centrifuged at 4,000 r.p.m. for 5 min and the pellet was re-suspended in 1 ml of fresh PBS and washed three times. Cultures were reconstituted to 1.0 OD_595 nm_, then diluted 1:1,000 and incubated with compounds **4**–**8** at the indicated concentrations (that is, concentrations matching the IC_50_ values in *A. fumigatus* for all compounds, except for compounds **3** and **5a** where a top concentration of 20 μM was used). Cell viability was monitored over 16 h by measuring OD_600 nm_ in a spectrophotometer. Data is represented as % of cell viability as means from at least two independent experiments with *n*=3.

### Determination of haemolytic activity

Erythrocytes were isolated from freshly drawn, anticoagulated human blood and diluted in PBS (1:5). An amount of 50 μl of erythrocyte suspension was added to 50 μl of compounds **4**–**8** at the indicated concentrations (that is, concentrations matching the IC_50_ values in *A. fumigatus* for all compounds, except for compounds **3** and **5a** where a top concentration of 20 μM was used). 0.2% Triton X-100 was used as positive control and PBS as negative control. The plate was incubated at 37 °C for 1 h, each well was diluted with 150 μl of PBS and the plate was centrifuged at 1,200*g* for 15 min. A total of 100 μl of the supernatant from each well was transferred to a fresh plate, and the absorbance at 350 nm was measured in a microplate reader. Data is represented as % of cell viability as means from three independent experiments with *n*=3.

### Confocal microscopy of *Aspergillus fumigatus* and human cells

Human lung A549 epithelial cells (ATCC CCL-185) were grown using DMEM supplemented with 10% fetal bovine serum (FBS), antibiotics (100 U ml^−1^ penicillin and 100 mg ml^−1^ streptomycin) and 2 mM L-glutamine in a humidified atmosphere at 37 °C with 5% CO_2_. A549 cells were regularly passaged in T-75 cell culture flasks. *A. fumigatus* was grown on standard Vogel's agar at 37 °C for 5 days. Conidia were collected using 0.05% Tween 80, re-suspended in 20% Vogel's liquid medium and incubated for 12 h at 25 °C. For co-cultures, human lung epithelial cells were plated on glass chamber slides Lab-Tek II (Nunc) 2 days before imaging and incubated for 16 h with *A. fumigatus* conidia reaching 75–90% confluence on the day of the experiment. For imaging experiments, cells were incubated for 15 min at 37 °C with compounds **5**–**8** (5 μM for compounds **5**–**7** and 2 μM for compound **8**) and imaged without washing in phenol red-free DMEM under a Zeiss LSM 510 META fluorescence confocal microscope equipped with a live cell imaging stage. Fluorescence and bright-field images were acquired using × 40 or × 63 oil objectives. Fluorescent probes were excited with 488 nm (compounds **5–8**) or 543 nm (Syto82) lasers. Confocal microscopy images were analysed and processed with ImageJ. Quantitative analysis of mean fluorescence intensities in competition experiments was performed with Imaris by calculating the mean intensity of each hyphae as independent regions of interests. For competition assays, all images were acquired and analysed using exactly the same conditions.

### Chemical stability in human bronchoalveolar lavages

Peptides **5** and **8** (20 μM) were dissolved in human bronchoalveolar lavage samples (total volume: 100 μl) and incubated at 37 °C for the indicated times. Samples were injected into an high-performance liquid chromatography Agilent 1100 separations module connected to a UV detector with a Discovery C_18_ column (5 μm, 4.6 × 50 mm). Matrix-assisted laser desorption/ionization data was recorded on a Bruker Ultraflex mass spectrometer using sinapinic acid as the matrix.

### Multi-photon imaging in *ex vivo* human tissue

*Ex vivo* human lung tissue experiments were approved by the NHS Lothian Tissue Governance Committee and Regional Ethics Committee (REC reference: ref. 13/ES/0126). Human lung tissue was obtained from the periphery (non-cancerous) region of patients undergoing resection for lung cancer. A 1 cm^3^ tissue was inflated with optimum cutting temperature formulation and stored at −80 °C. Embedded tissue was cryosectioned at 10 μm intervals and fixed onto glass slides for imaging. RFP-expressing *A. fumigatus* conidia were grown overnight at 37 °C the day before the experiments and incubated with human lung tissue sections for 2–3 h before imaging. For multi-photon imaging experiments, the cyclic peptide **8** was used at a concentration of 5 μΜ. A custom-built multi-photon microscope was used to acquire second harmonic generation (SHG) and two-photon fluorescence images. Briefly, a picoEmerald (APE) laser provided both a tunable pump laser (720–990 nm, 7 ps, 80 MHz repetition rate) and a spatially overlapped Stokes laser (1064, nm, 5–6 ps and 80 MHz repetition rate). GFP two-photon fluorescence signals were filtered using the following series of filters: FF520-Di02, FF483/639-Di01 and ET500/40m. RFP two-photon fluorescence signals were filtered using FF520-Di02, FF757-Di01 and FF01-609/181, and SHG signals were filtered using FF520-Di02, FF483/639-Di01 and FF01-466/40. Fluorescence lifetime images were acquired by connecting the relevant detector to a PicoHarp 300 (Picoquant, Berlin) and configuring the PMT for photon counting mode for TCSPC-FLIM. SHG and GFP images were taken with the laser tuned to 950 nm and RFP images were recorded using a 1,064 nm laser. Lifetime images were recorded at 20 mW with a 10 μs pixel dwell using the SymPhoTime software (Picoquant). All images were analysed and processed using ImageJ.

## Additional information

**How to cite this article:** Mendive-Tapia, L. *et al.* Spacer-free BODIPY fluorogens in antimicrobial peptides for direct imaging of fungal infection in human tissue. *Nat. Commun.* 7:10940 doi: 10.1038/ncomms10940 (2016).

## Supplementary Material

Supplementary Information Supplementary Figures 1-25, Supplementary Discussion, Supplementary Methods and Supplementary References

Supplementary Movie 1 3D projection of fluorescence images of A. fumigatus after incubation with the peptide 8. Peptide 8 (2 μM) was incubated for 15 min in A. fumigatus that had been not pretreated with PAF26, and cells were imaged under a confocal microscope at 37 °C. Scale bar: 10 μm.

Supplementary Movie 2 3D projection of fluorescence images of PAF26 pre-treated A. fumigatus after incubation with the peptide 8. Peptide 8 (2 μM) was incubated for 15 min in A. fumigatus that had been pre-treated with PAF26 (3 μM) for 30 min, and cells were imaged under a confocal microscope at 37 °C. Scale bar: 10 μm.

Supplementary Movie 3 Time-lapse high-resolution imaging of A. fumigatus upon treatment with the peptide 8. A. fumigatus were pre-treated with a cell membrane counterstain (red signal) and imaged under confocal microscope. After 20 s, cells were treated with the peptide 8 (2 μM, green signal) and further imaged without any washing. The movie shows the rapid fluorogenic response of 8 upon interaction with the fungal cell membrane and subsequent internalisation in lipophilic environments. Scale bar: 2.5 μm.
