# Supplementary material for: Spacer-free BODIPY fluorogens in antimicrobial peptides for direct imaging of fungal infection in human tissue
Source: Nat Commun. 2016 Mar 9;7:10940. doi: 10.1038/ncomms10940 (PMC4786873; doi:10.1038/ncomms10940)
Supplement: Supplementary Information — Supplementary Figures 1-25, Supplementary Discussion, Supplementary Methods and Supplementary References [file ncomms10940-s1.pdf]

Supplementary Figures

Spectral properties of compound 3.

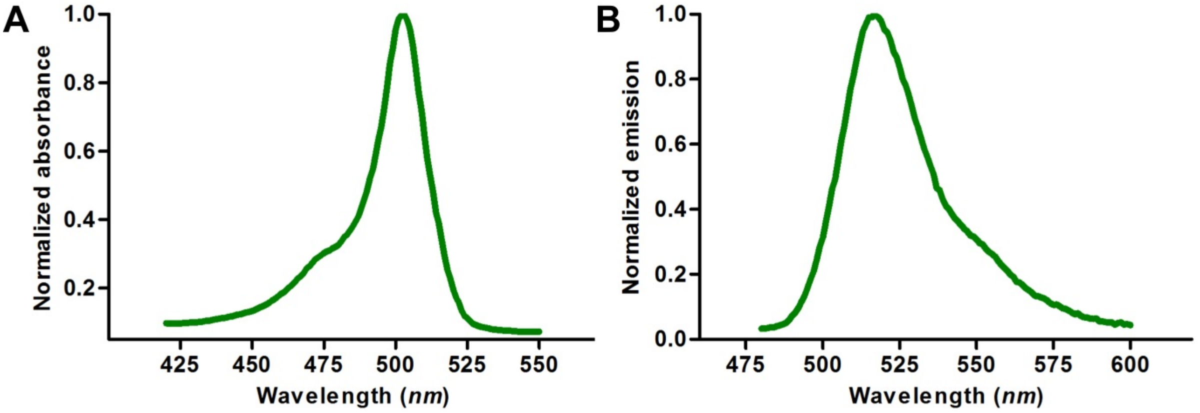

Supplementary Figure 1. Spectral characterisation of the amino acid 3. A) Absorbance and B) emission spectra ($\lambda_{exc.}$: 450 nm) of compound **3**.

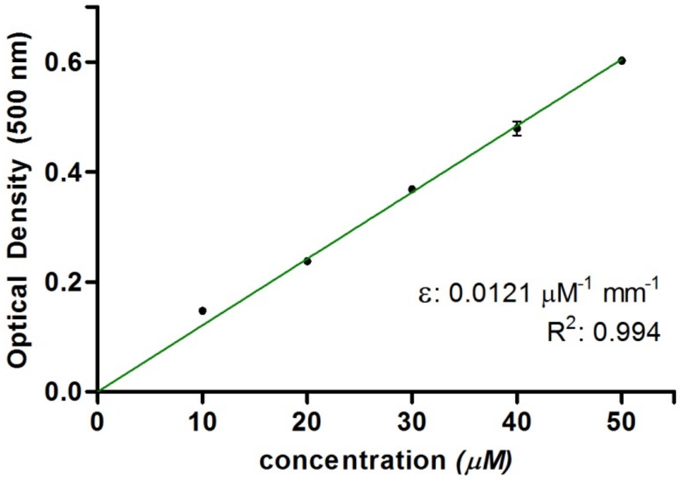

Supplementary Figure 2. Determination of the extinction coefficient of the amino acid 3. Solutions of **3** were prepared in ethanol and their optical densities were measured at 500 nm in a NanoDrop 1000 spectrophotometer. Data represented as means ± s.e.m. (*n*=3).

Molecular simulation models of labelled and non-labelled peptides.

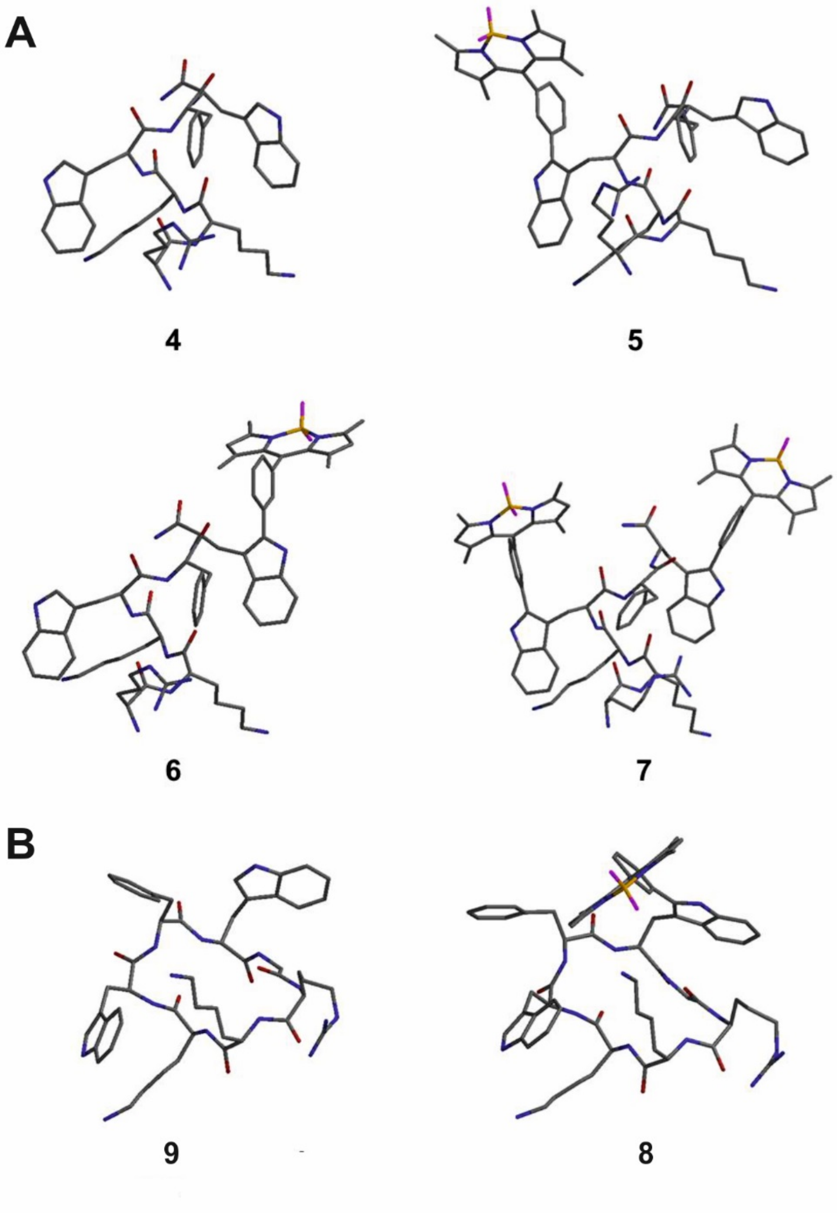

Supplementary Figure 3. A) Optimised geometries of fluorogenic linear peptides **5**-**7** and the corresponding non-labelled linear peptide **4**. B) Optimised geometries of the fluorogenic cyclic peptide **8** and the corresponding non-labelled cyclic peptide **9** (for structure see Supplementary Fig. 8). All simulations were generated by the Spartan '14 suite using Molecular Mechanics (MMFF94) and Semi-Empirical (AM1) methods.*

* Spartan'14 for Windows, Macintosh and Linux, version 1.1.4, wavefunction, inc. www.wavefun.com.

Antimicrobial and haemolysis assays.

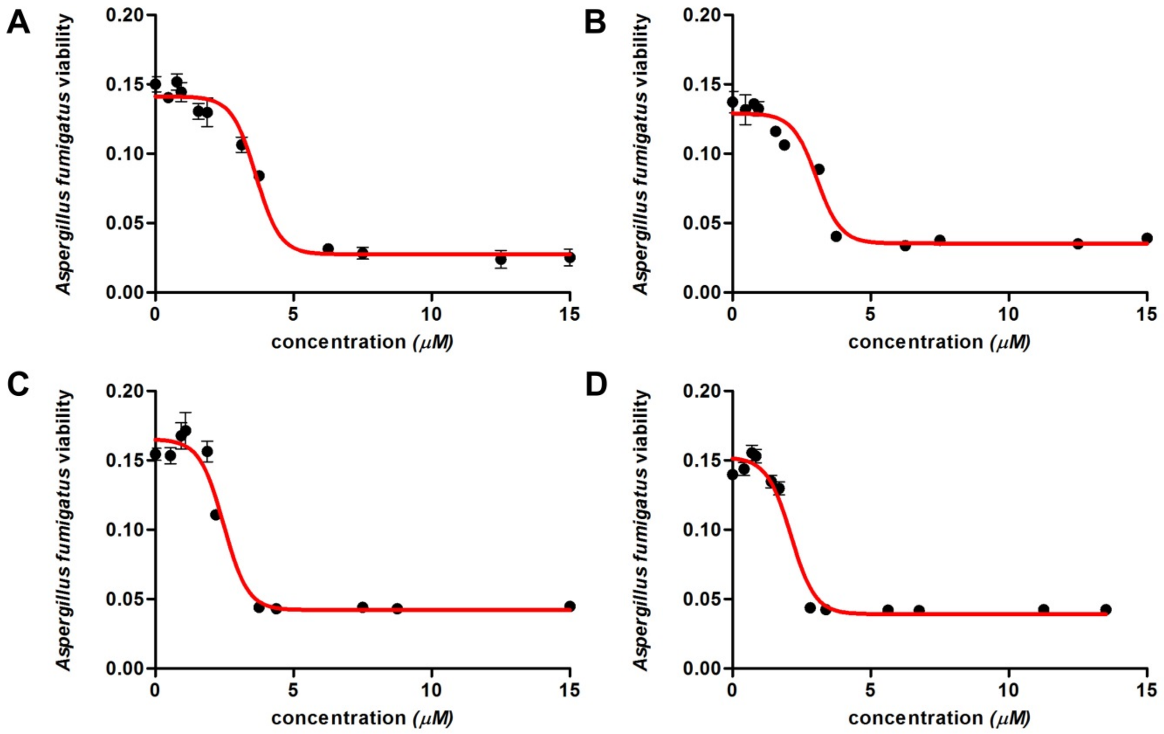

Supplementary Figure 4. Determination of IC_{50} values of BODIPY-labelled peptides 5-8 in *A. fumigatus*. Cell viability plots and non-linear regressions for peptides **5** (A), **6** (B), **7** (C) and **8** (D). Peptides were incubated at different concentrations with *A. fumigatus* conidia to reach a final volume of 100 μL per well. The final conidia concentration was 5 × 10^5 cells/mL in 10% Vogel's medium. After 24 h incubation at 37 ºC in 96 well-plates, fungal growth was determined by measuring the optical density at 610 nm. The IC_{50} values were determined using four parameter logistic regression. Data represented as means ± s.e.m (*n*=3).

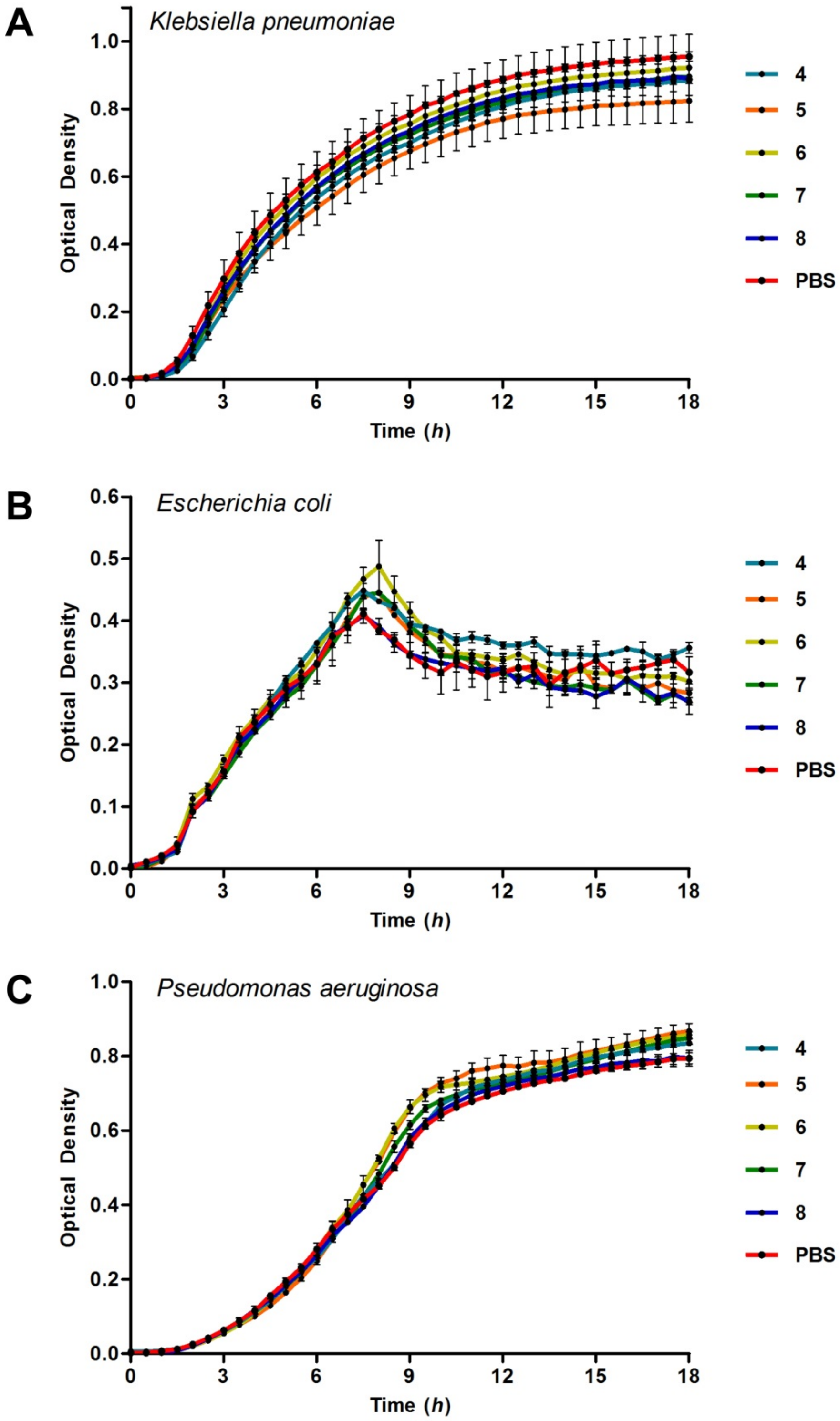

Supplementary Figure 5. Activity of peptides 4-8 in different bacterial strains. Peptides were added to the *Klebsiella pneumoniae* (A), *Escherichia coli* (B) and *Pseudomonas aeruginosa* (C), and their viability (optical density at 595 nm) was monitored at 37 ºC for 16 h. Data represented as means ± s.d. (*n*=3).

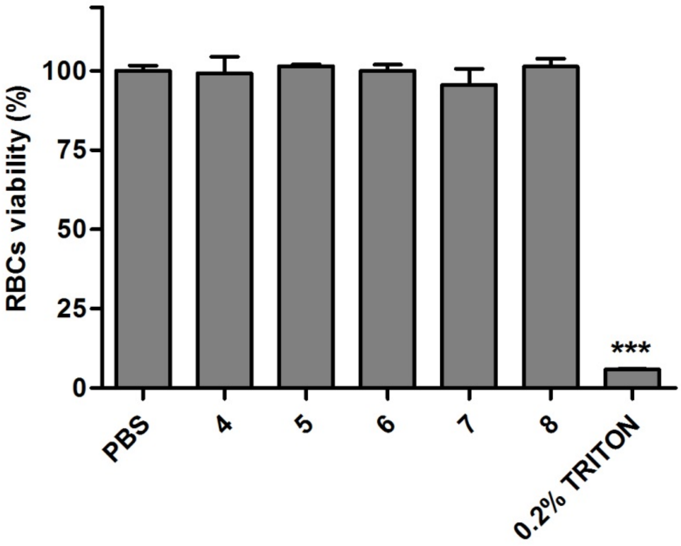

Supplementary Figure 6. Haemolytic activity of peptides 4-8 in human red blood cells (RBCs). Peptides **4**-**8** and Triton X-100 (0.2% as positive control) were incubated with human RBCs at 37 ºC and their viability was assessed after 1 h. Data represented as means ± s.d. (n=3). *** for p < 0.001 was determined as a statistically significant difference between the cell viabilities in PBS (phosphate buffer saline) and in Triton X-100.

Spectral properties of peptides 5-7.

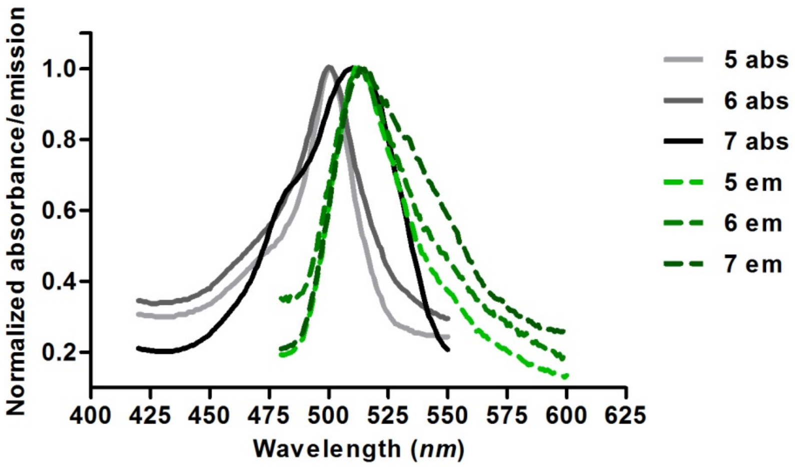

Supplementary Figure 7. Spectral characterisation of peptides 5, 6 and 7. Absorbance (solid lines) and emission (dashed lines) spectra ($\lambda_{exc.}$: 450 nm) of peptides **5**-**7** in PBS (phosphate buffer saline).

Characterisation of compounds 3, 5a, 5b and 9.

a

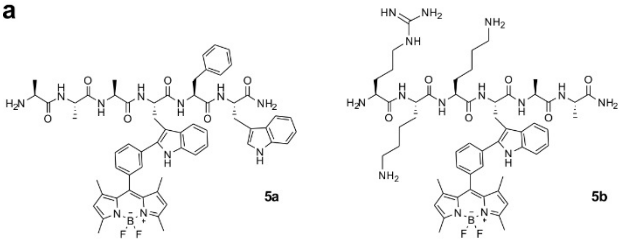

b

	Aspergillus fumigatus[1]	*Klebsiella pneumoniae*[2]	*Escherichia coli*[2]	*Pseudomonas aeruginosa*[2]	Human RBCs[3]
**3**	> 50	94%	>99%	>99%	99%
**5a**	> 50	70%	>99%	87%	99%
**5b**	4.6 ± 0.4	99%	>99%	94%	>99%
**9**	7.7 ± 0.7	97%	>99%	84%	99%

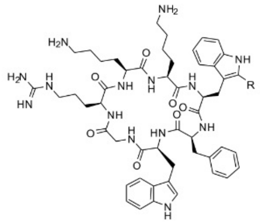

c

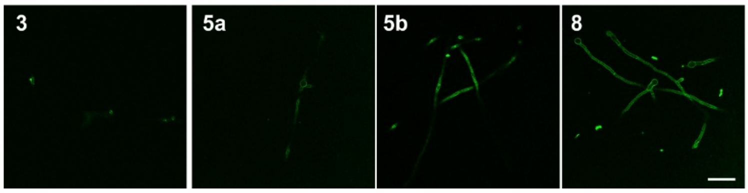

Supplementary Figure 8. Characterisation of the amino acid 3 and peptides 5a, 5b and 9 for live cell imaging of *A. fumigatus*. a) Chemical structures of fluorogenic linear (**5a**, **5b**) and cyclic peptides (**8**, **9**). b) Activity in *A. fumigatus*, several bacterial strains and in human red blood cells (RBCs).[1] IC_{50} (μM) values as means ± s.e.m. (*n*=3), [2] cell viability upon 16 h incubation with **3**, **5a**, **5b** or **9** at their respective IC_{50} concentrations or 20 μM for compounds **3** and **5a** (*n*=3), [3] cell viability upon 1 h incubation with **3**, **5a**, **5b** or **9** at their respective IC_{50} concentrations or 20 μM for compounds **3** and **5a** (*n*=3). c) Wash-free fluorescent live cell images of *A. fumigatus* at 37 ºC using confocal microscopy after incubation with peptides with **3**, **5a**, **5b** or **8** (all at 2 μM). Scale bar: 20 μm.

Stability assays.

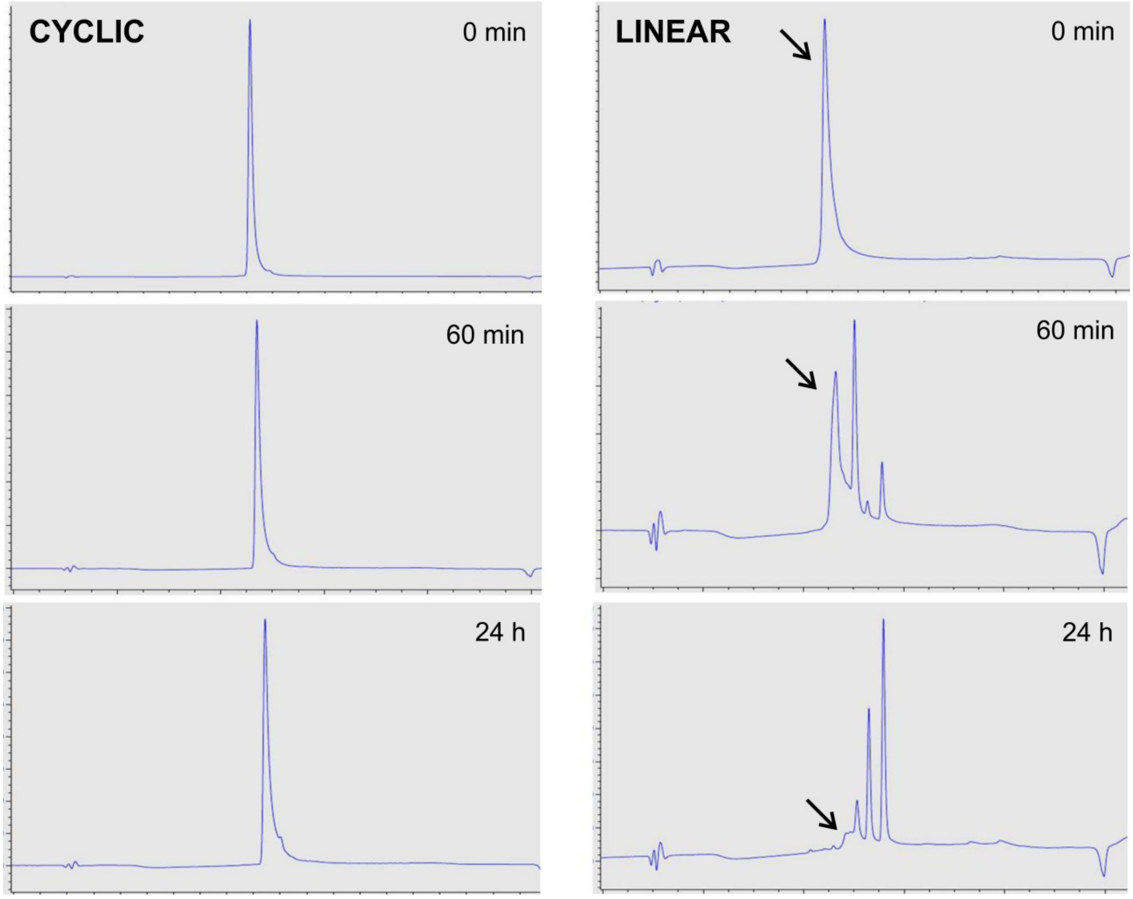

Supplementary Figure 9. Stability of cyclic (8) and linear (5) mono-BODIPY labelled peptides in human bronchoalveolar lavage samples from patients with acute respiratory distress syndrome. Peptides were incubated in human bronchoalveolar lavage samples at 37 ºC and analysed by HPLC at the time points indicated. Arrows point at the peaks corresponding to the intact linear peptide.

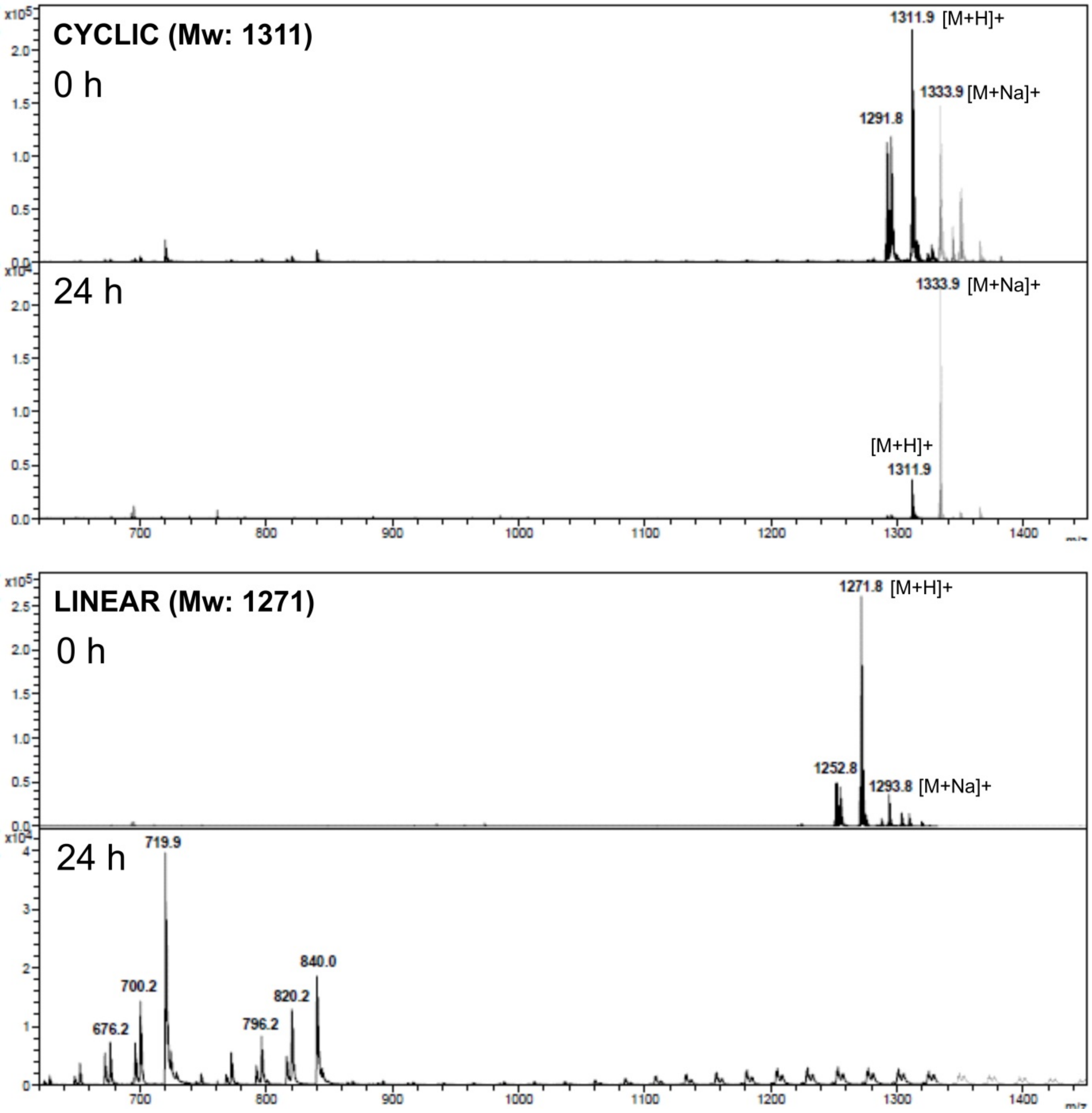

Supplementary Figure 10. MALDI analysis of cyclic (8) and linear (5) mono-BODIPY labelled peptides in bronchoalveolar lavage samples from patients with acute respiratory distress syndrome. Peptides were incubated in human bronchoalveolar lavage samples at 37 ºC and analysed by MALDI at the time points indicated.

Synthesis and spectral properties of peptide 8.

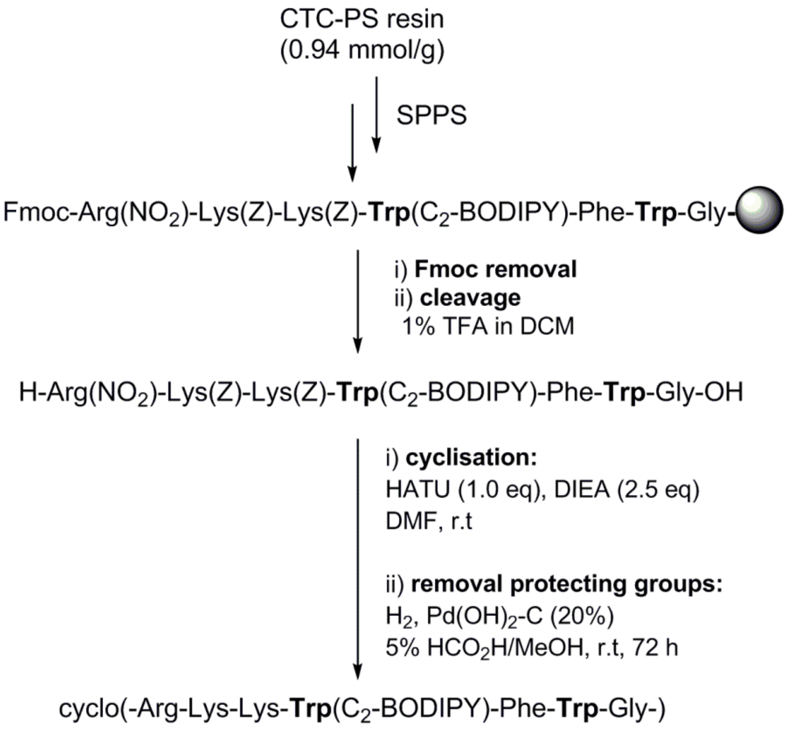

Supplementary Figure 11. Synthetic scheme for the cyclic peptide **8**.

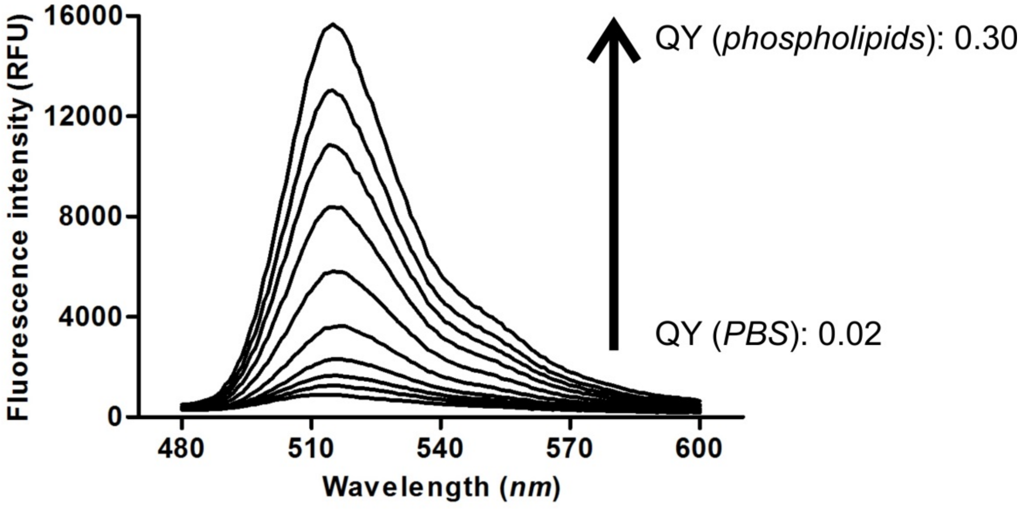

Supplementary Figure 12. Fluorogenic behaviour of the cyclic peptide 8 in phospholipid membranes. Spectra of peptide **8** were recorded after incubation with phosphatidylcholine:cholesterol (7:1) liposomes suspensions in phosphate buffer saline (PBS) ranging from 3.75 mg mL^{-1} to 0.004 mg mL^{-1} of PC in 2-fold serial dilutions, $\lambda_{exc.}$: 450 nm. Quantum yields were determined using fluorescein in basic ethanol as the reference (QY: 0.97).[1]

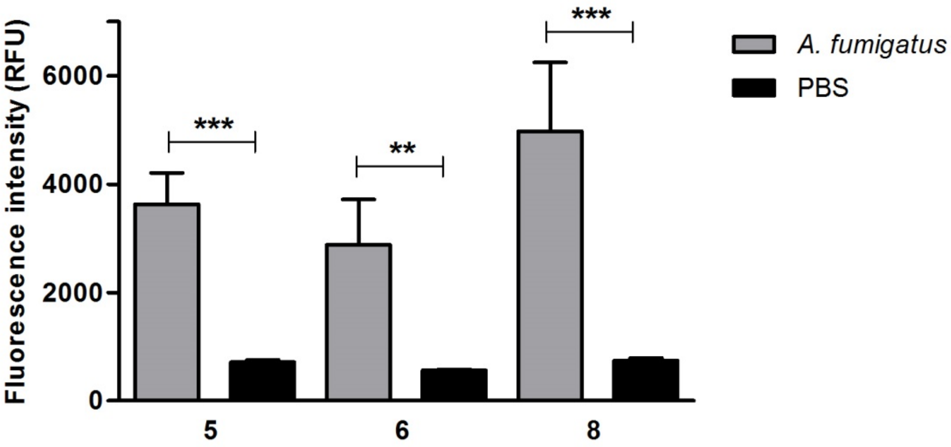

Supplementary Figure 13. Fluorescence emission of mono-BODIPY labelled peptides 5, 6 (linear) and 8 (cyclic) upon incubation with *A. fumigatus*. Peptides **5**, **6**, and **8** were incubated in PBS (phosphate buffer saline) alone or in suspensions of *A. fumigatus* in PBS ($\lambda_{exc.}$: 485 nm; $\lambda_{em.}$: 515 nm). Data represented as means ± s.d. (n=3). ** for $p < 0.01$ and *** for $p < 0.005$ were determined as statistically significant differences between the fluorescence emission values in PBS and in suspensions of *A. fumigatus* in PBS.

Confocal microscopy images of different fungal species.

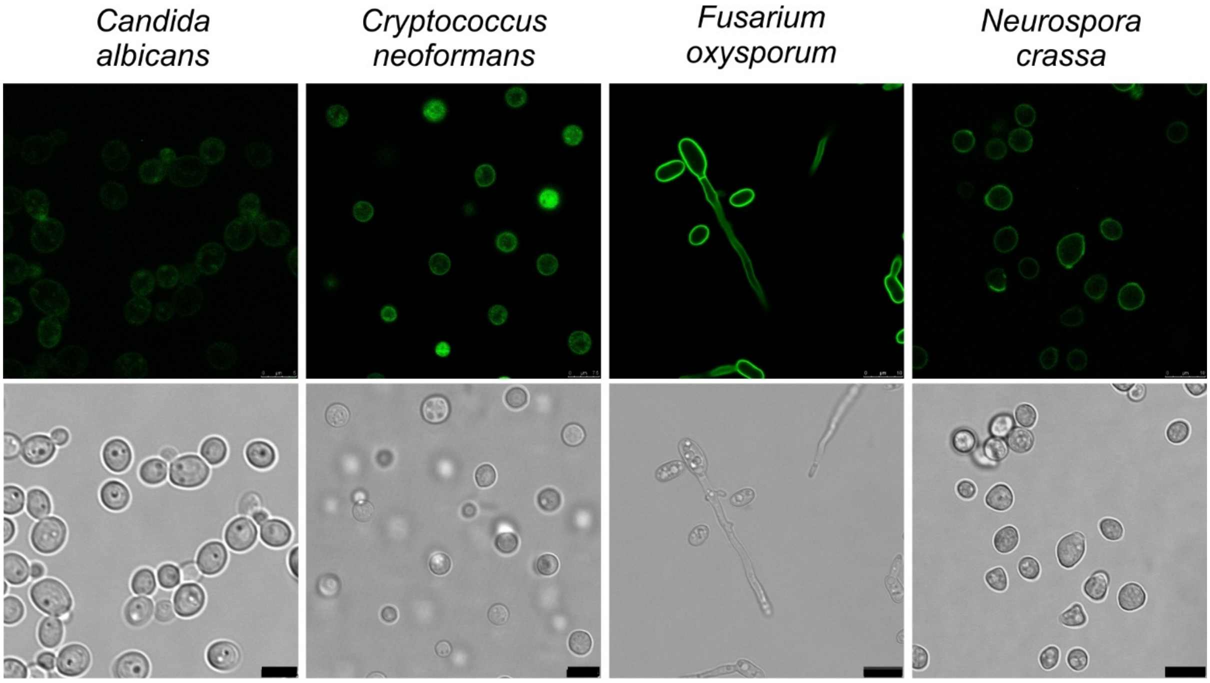

Supplementary Figure 14. Live cell imaging of fungal cells after incubation with the peptide 8. Peptide **8** was used at 2 μM for all fungi except *C. albicans* for which 10 μM was used. Fluorescence (*top*) and correspondinding brightfield (*bottom*) images were acquired under a confocal microscope at r.t.. Scale bars: *C. albicans*: 5 μm; *C. neoformans*: 7.5 μm; *F. oxysporum* and *N. crassa*: 10 μm.

Competition experiments between 8 and PAF26 (4).

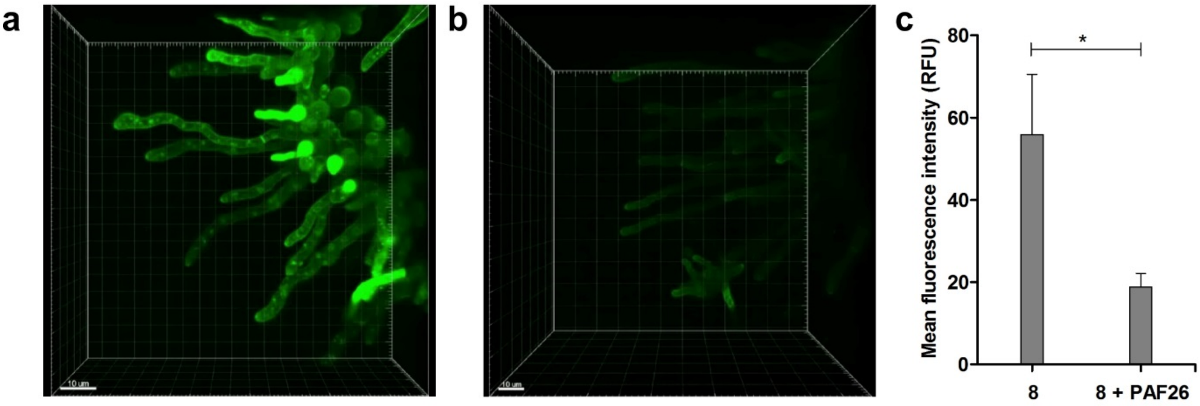

Supplementary Figure 15. Fluorescence images of *A. fumigatus* after incubation with the peptide 8 without and with pre-treatment of PAF26 (4). Peptide **8** (2 μM) was incubated for 15 min with *A. fumigatus* that had not been pre-treated (a) or had been pre-treated with PAF26 (3 μM) for 30 min at 37°C (b). Images were captured under a confocal microscope at r.t. For 3D projections, see Supplementary Movies 1 and 2. Scale bars: 10 μm. c) Mean fluorescence intensity values were determined using the software Imaris and represented as means ± s.d. (n=3).* for p < 0.05 was determined as a statistically significant difference between PAF26 pre-treated and not pre-treated *A. fumigatus*.

Confocal microscopy images of *Aspergillus fumigatus*.

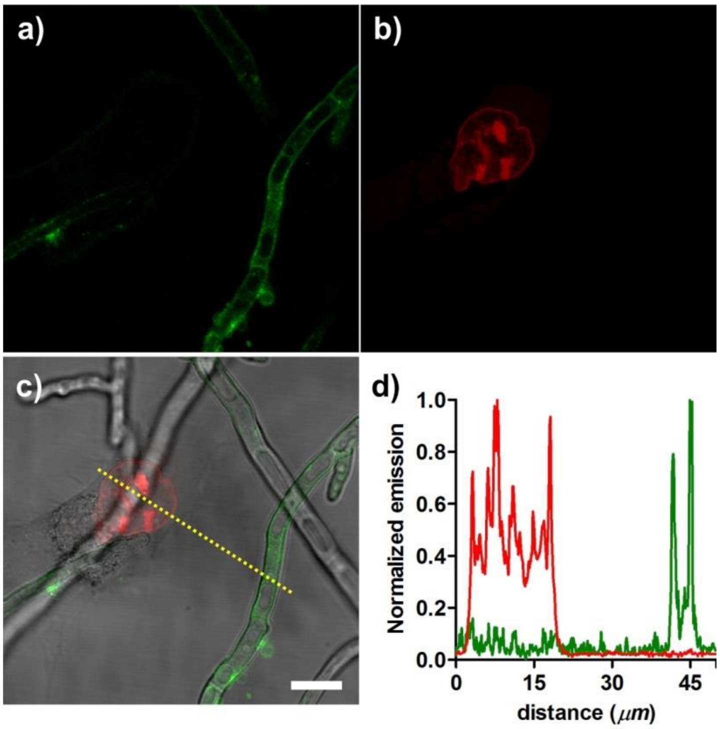

Supplementary Figure 16. Live cell imaging of *A. fumigatus* in co-cultures with human lung A549 epithelial cells after treatment with the peptide 8. Peptide **8** (2 μM, green) and Syto82 (2.5 μM, red counterstain for human lung cells) were incubated in co-cultures and imaged without washing under a confocal microscope at 37 ºC. Fluorescence staining of **8** (a), Syto82 (b) and merged (c). (d) Plot profile analysis of the staining from **8** (green) and Syto82 (red) shown in the merged image. Scale bar: 10 μm.

Cell viability assays.

Cell viability was determined with a TACS® MTT Cell Proliferation assay (Trevigen) according to the manufacturer's instructions. Briefly, human lung A549 epithelial cells were plated on 96-well plates the day before the experiment, reaching 90-95% confluence on the day of the experiment. The peptide **8** was added to the cells at different concentrations (0, 1.25, 2.5, 5 and 10 μM) and incubated at 37 ºC for 4 h. After 4 h, cells were washed, treated according to the manufacturer's instructions and their absorbance values (570 nm) were measured in a Synergy HT spectrophotometer (Biotek). Cell viability data was normalised to the proliferation of cells without addition of the peptide **8**.

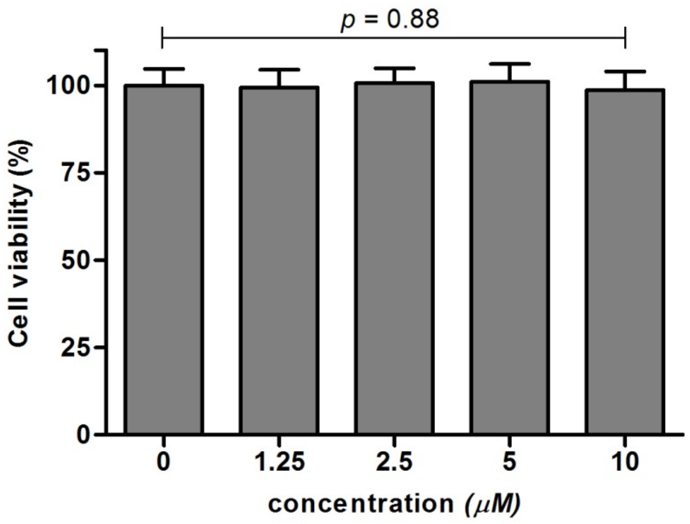

Supplementary Figure 17. Cell proliferation assays. Viability of human lung A549 epithelial cells after incubation with different concentrations of the peptide **8**. Data represented as means ± s.d. (n=4). No significant differences ($p > 0.05$) were determined between the control and any of the treatments.

Chemical characterisation data.

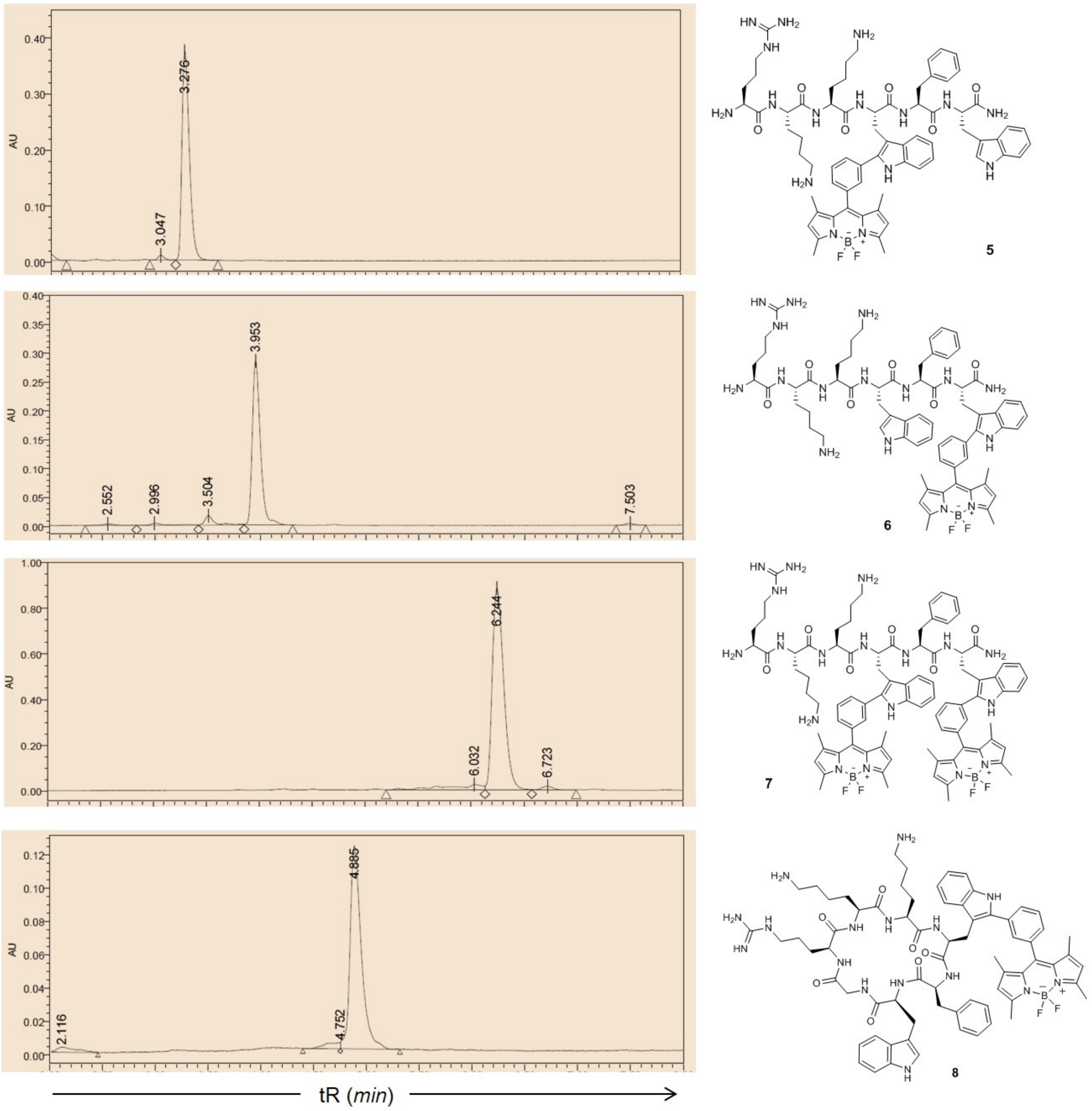

Supplementary Figure 18. HPLC analysis of BODIPY-labelled peptides **5-8**.

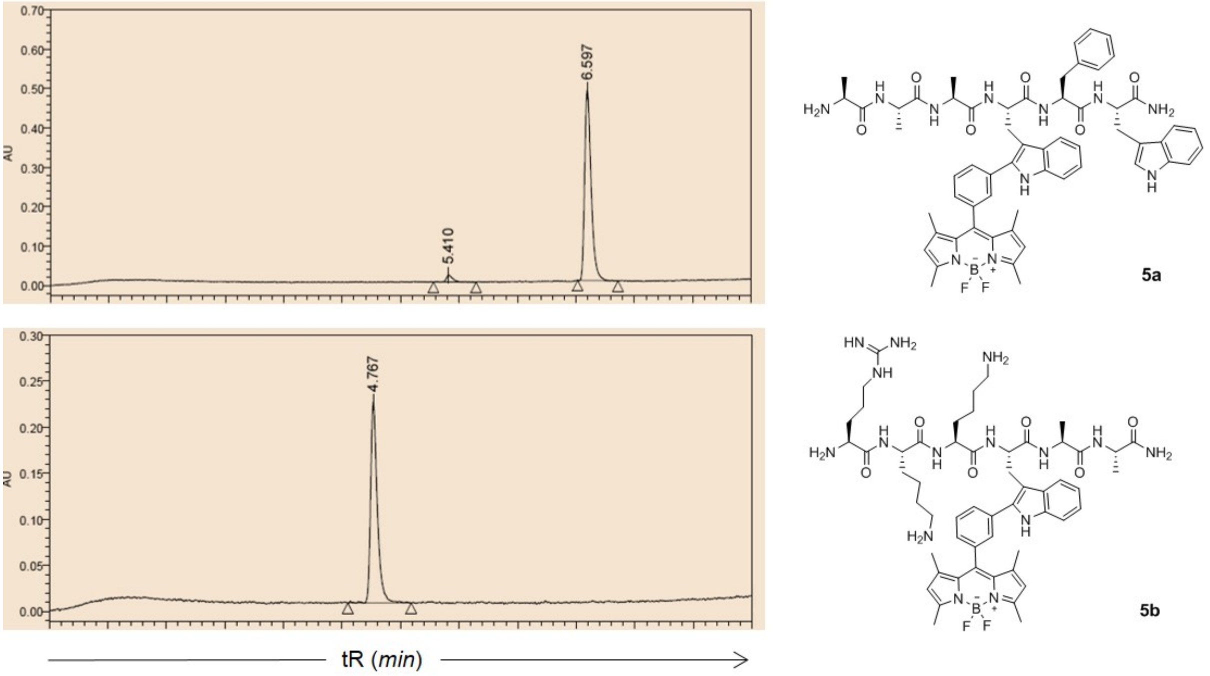

Supplementary Figure 19. HPLC analysis of BODIPY-labelled peptides **5a** and **5b.**

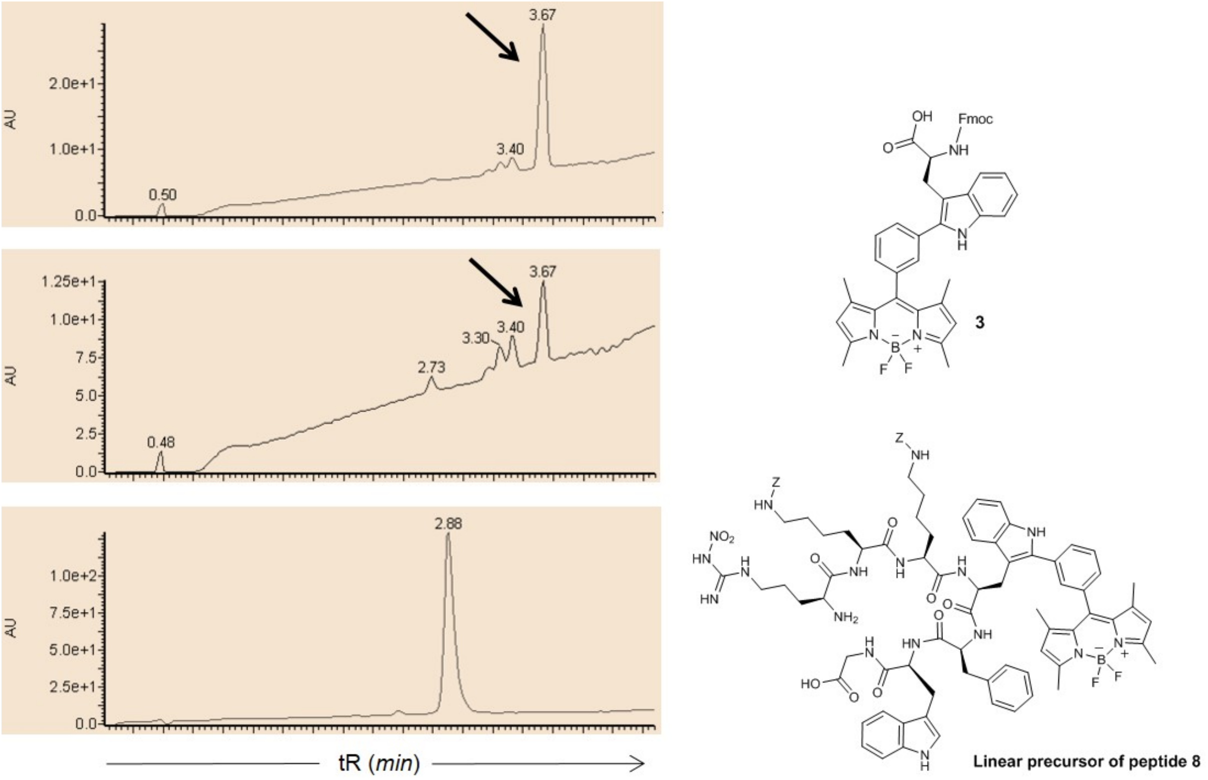

Supplementary Figure 20. Stability studies of BODIPY derivatives. HPLC analysis of Fmoc-Trp(C_2-BODIPY)-OH (**3**) in TFA:DCM (1:99) at r.t. after 10 min (83% purity) (*top panel*), Fmoc-Trp(C_2-BODIPY)-OH (**3**) in in TFA:DCM (1:9) at r.t. after 30 min (46% purity) (*mid panel*), crude BODIPY-labelled linear precursor of the cyclic peptide **8** directly after cleavage from the resin with TFA:DCM (1:99) at r.t. (*bottom panel*) (98% purity). Black arrows in top and mid panels point at the peak corresponding to the amino acid **3**.

^1H NMR ($CDCl_3$)

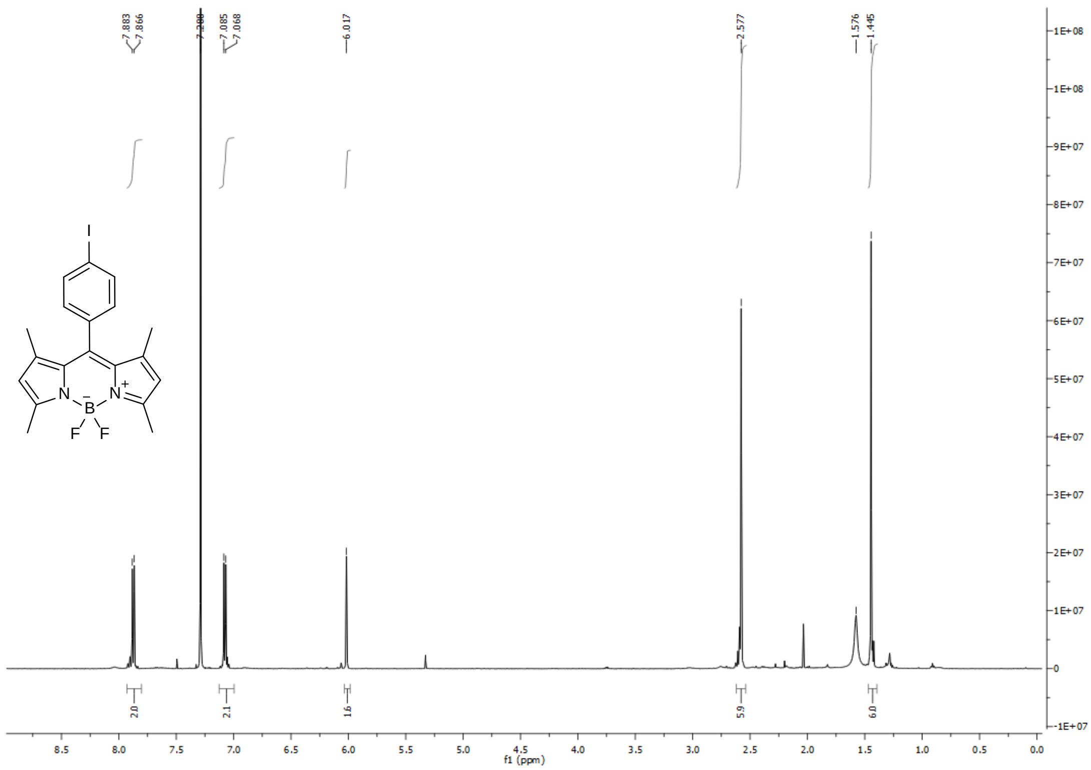

^{13}C NMR ($CDCl_3$)

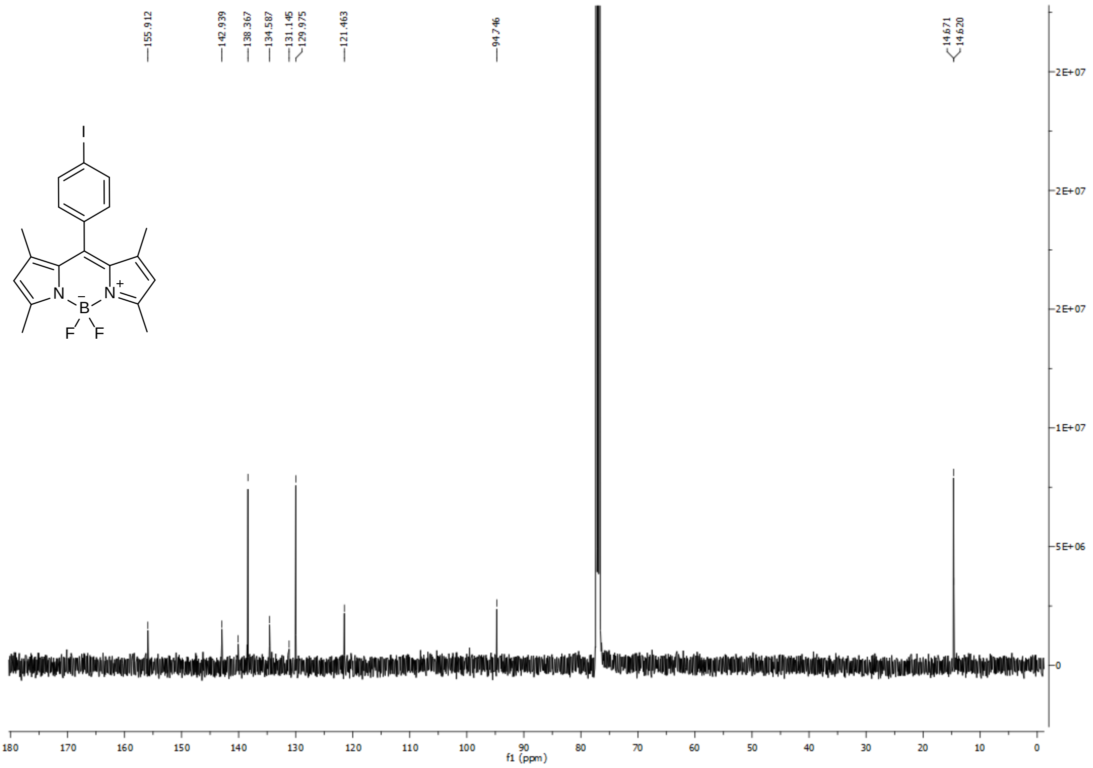

Supplementary Figure 21. NMR characterisation of 4,4-difluoro-8-(4-iodophenyl)-3,5-dimethyl-4-bora-*3a,4a*-diaza-*s*-indacene (**1**).

^{1}H NMR ($CDCl_3$)

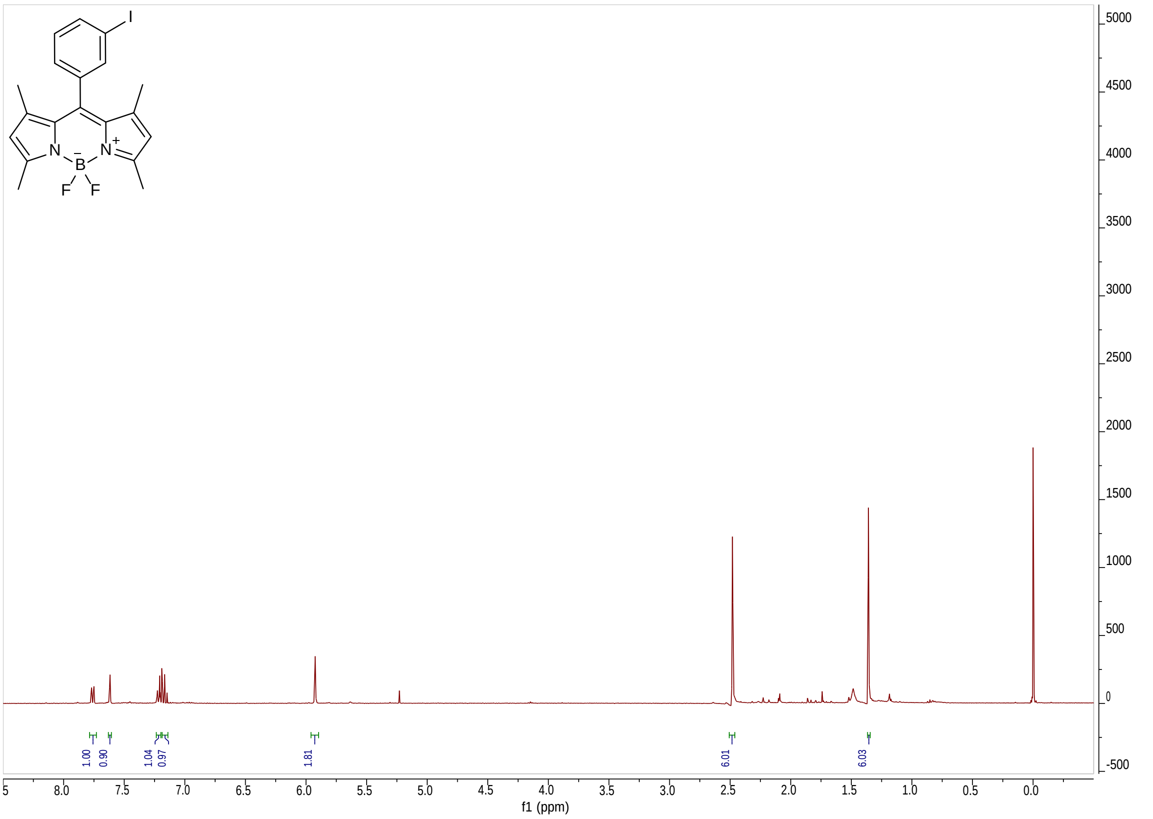

^{13}C NMR ($CDCl_3$)

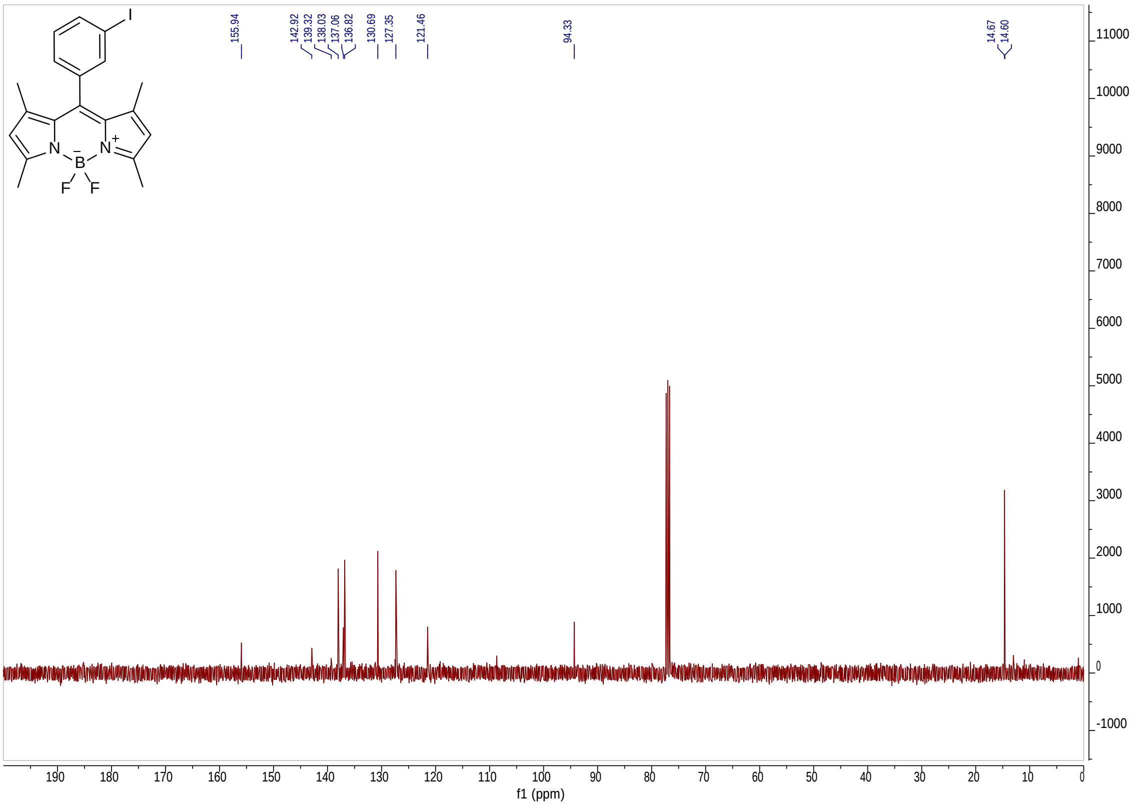

Supplementary Figure 22. NMR characterisation of 4,4-difluoro-8-(3-iodophenyl)-3,5-dimethyl-4-bora-*3a*,*4a*-diaza-*s*-indacene (**2**).

^1H NMR ($CDCl_3$)

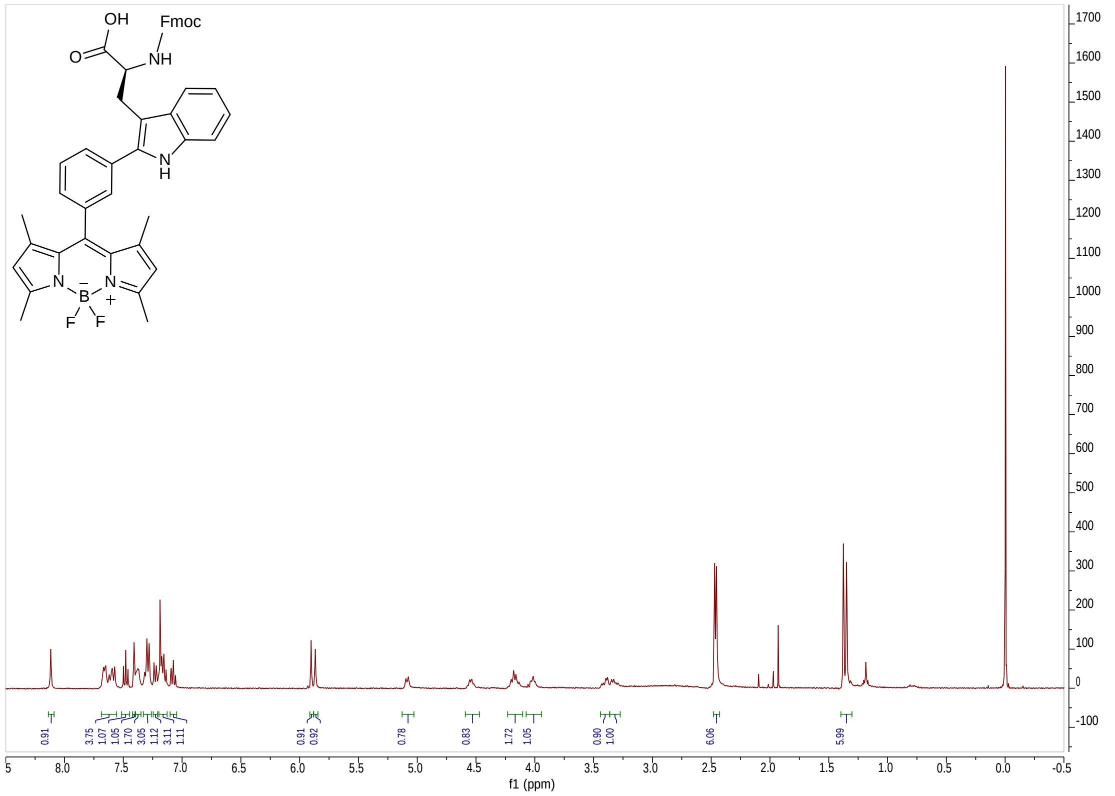

^{13}C NMR ($CDCl_3$)

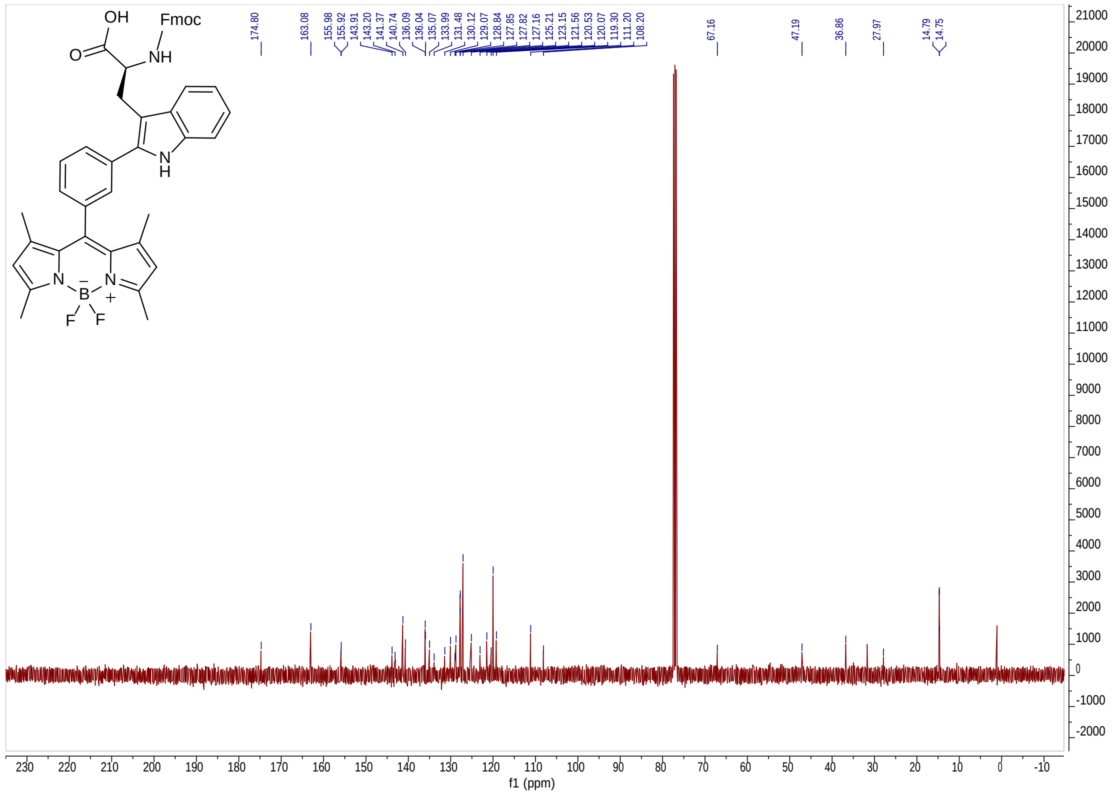

Supplementary Figure 23. NMR characterisation of Fmoc-Trp(C_2-BODIPY)-OH (**3**).

^{1}H NMR (MeOD)

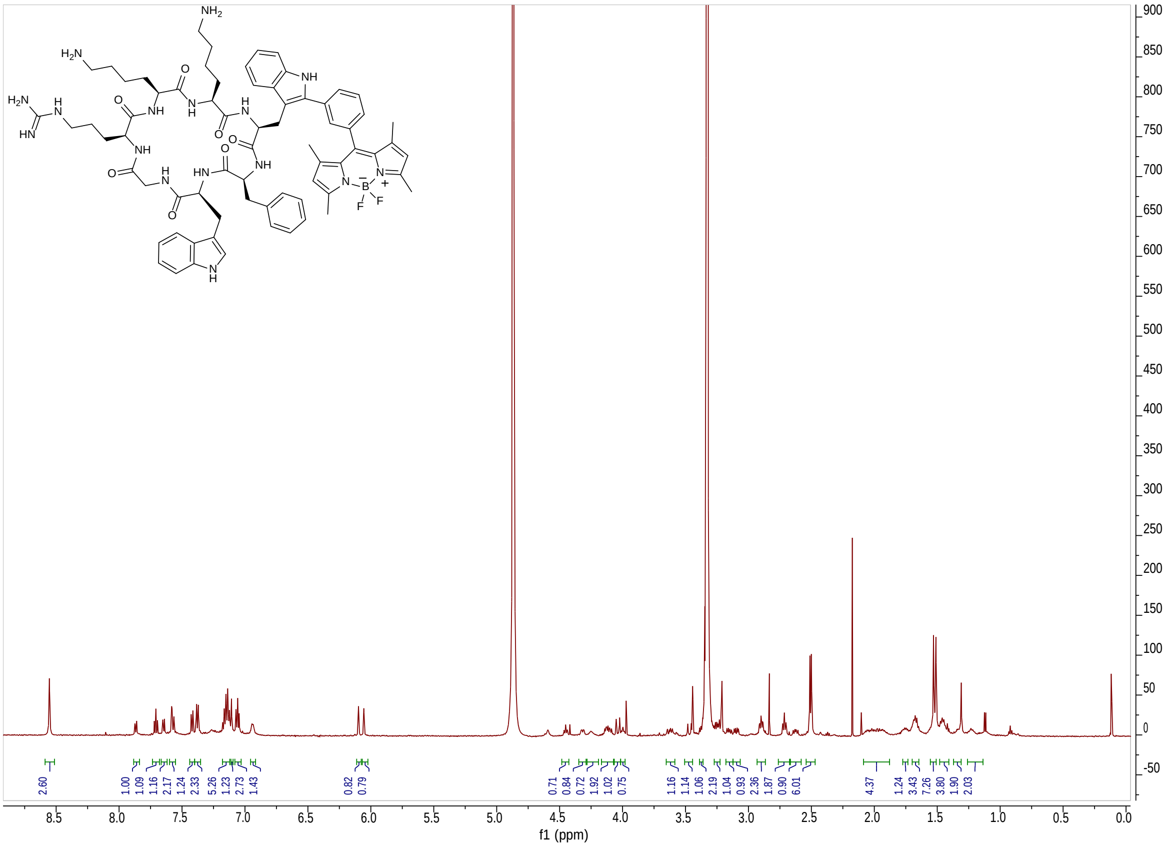

HSQC NMR (MeOD)

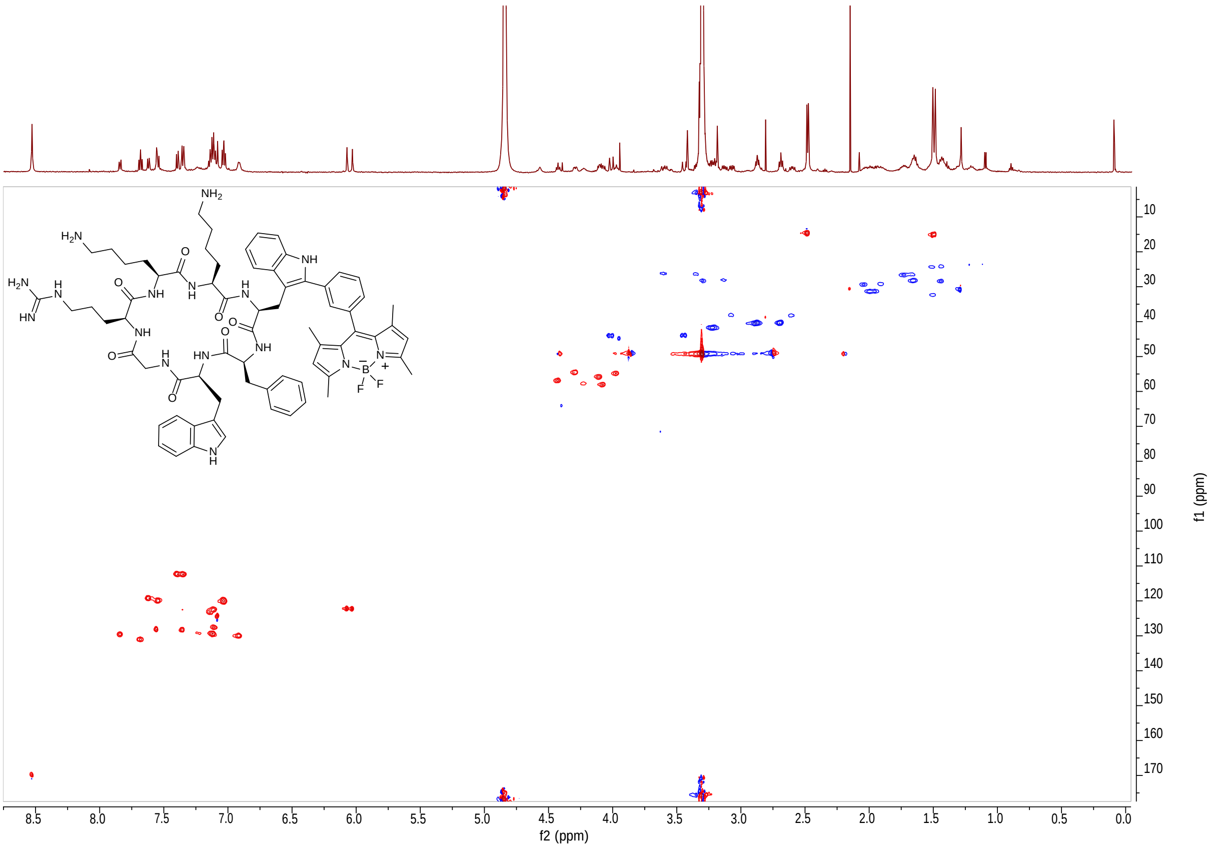

COSY NMR (MeOD)

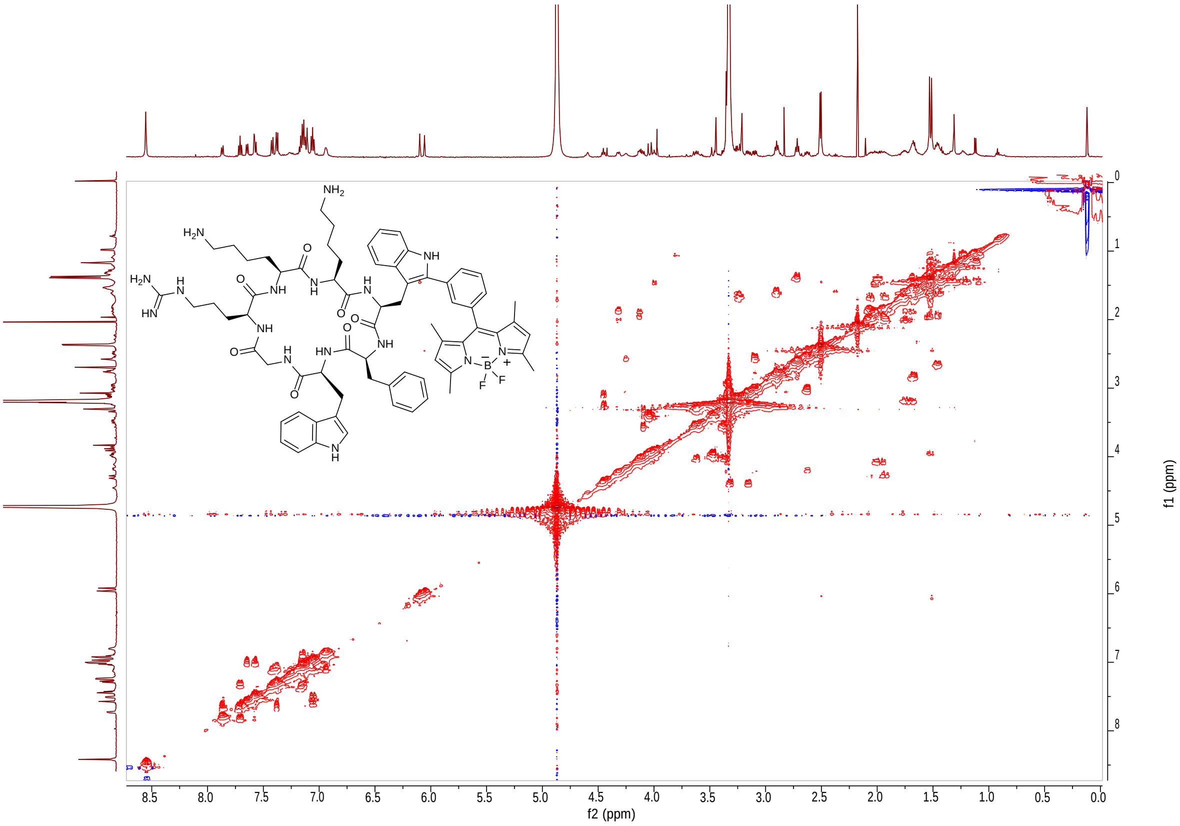

TOCSY NMR (MeOD)

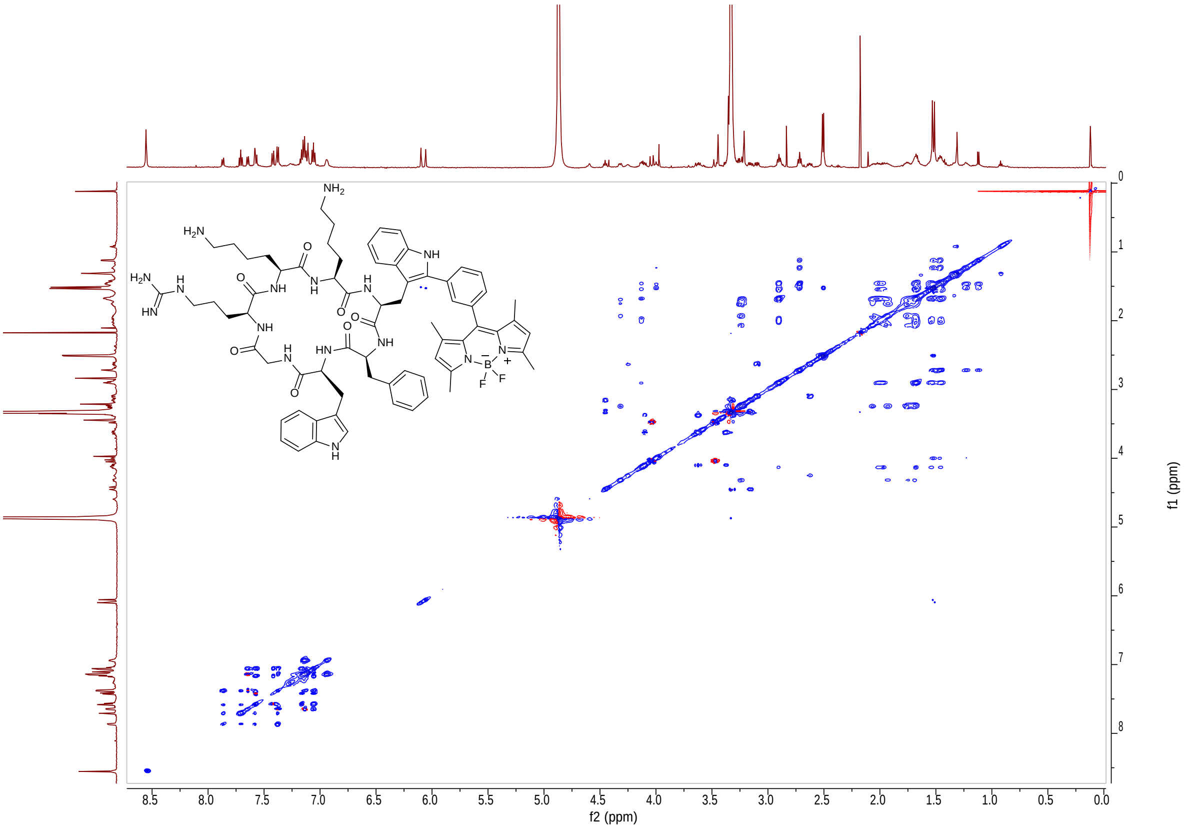

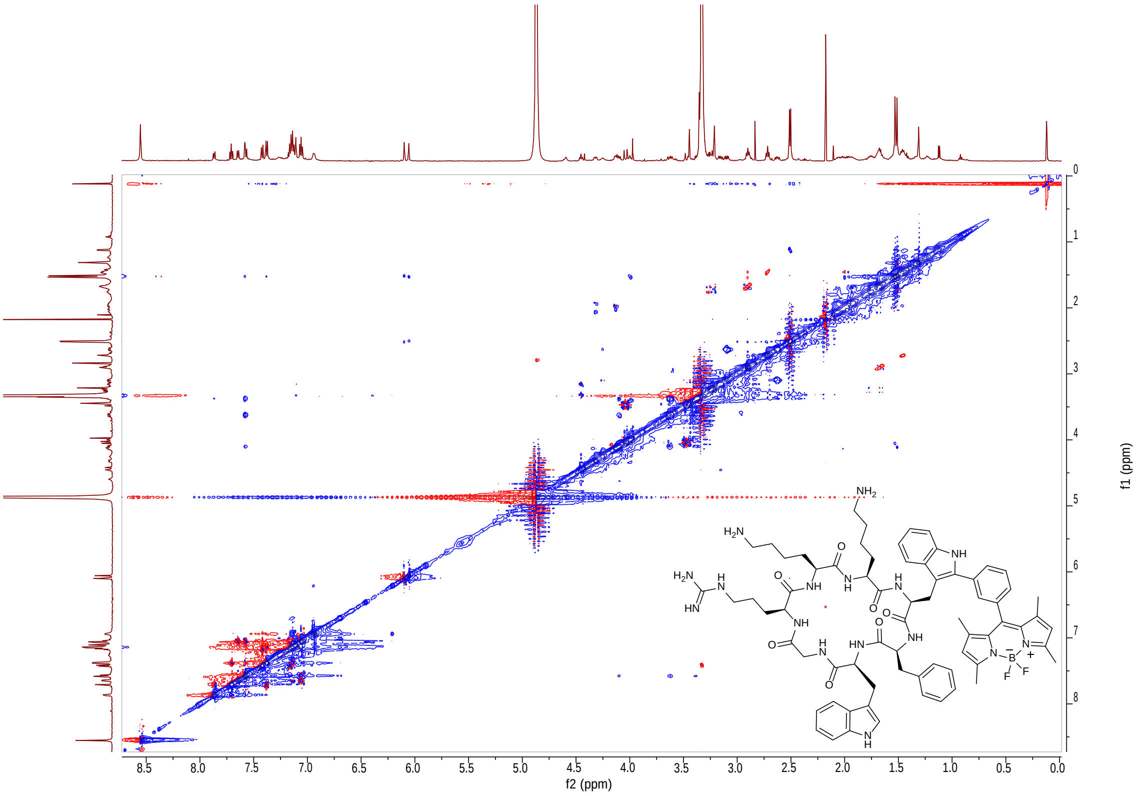

Supplementary Figure 24. NMR characterisation of cyclo(Arg-Lys-Lys-Trp(C_2-BODIPY)-Phe-Trp-Gly) (**8**).

^{1}H-NMR (DMSO-*d6*)

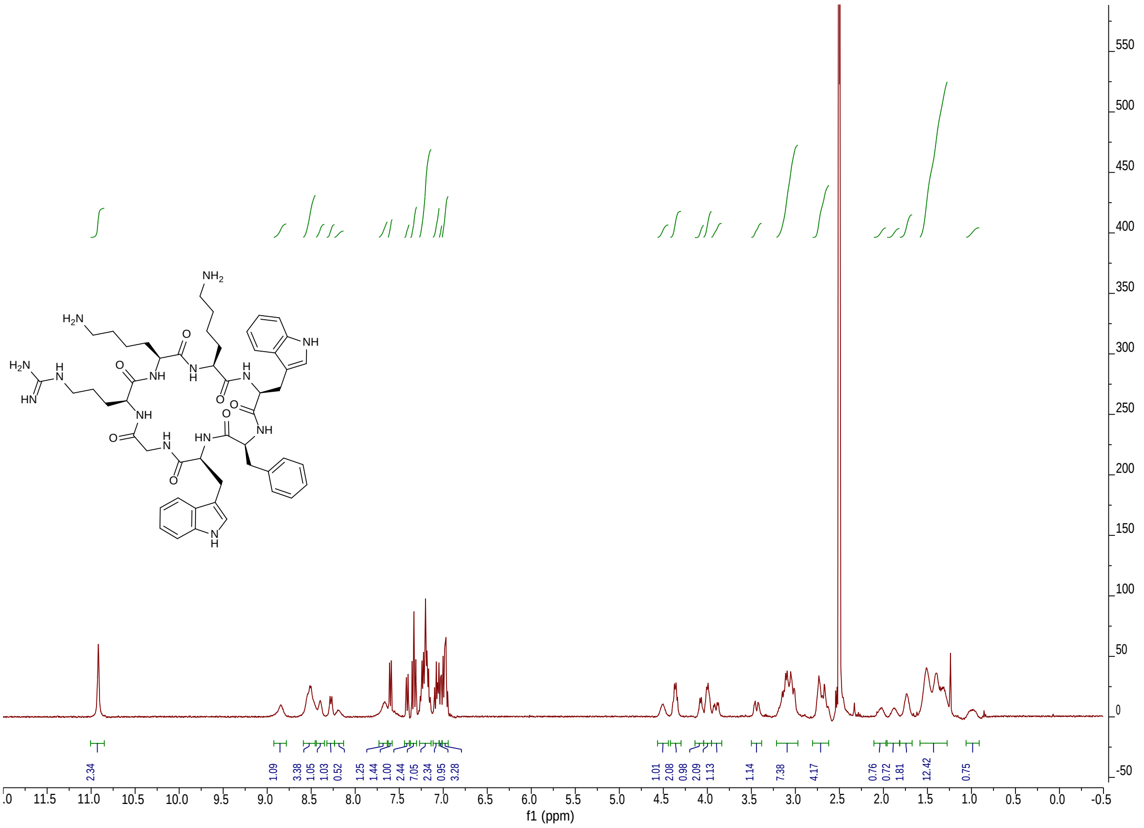

HSQC NMR (DMSO-*d6*)

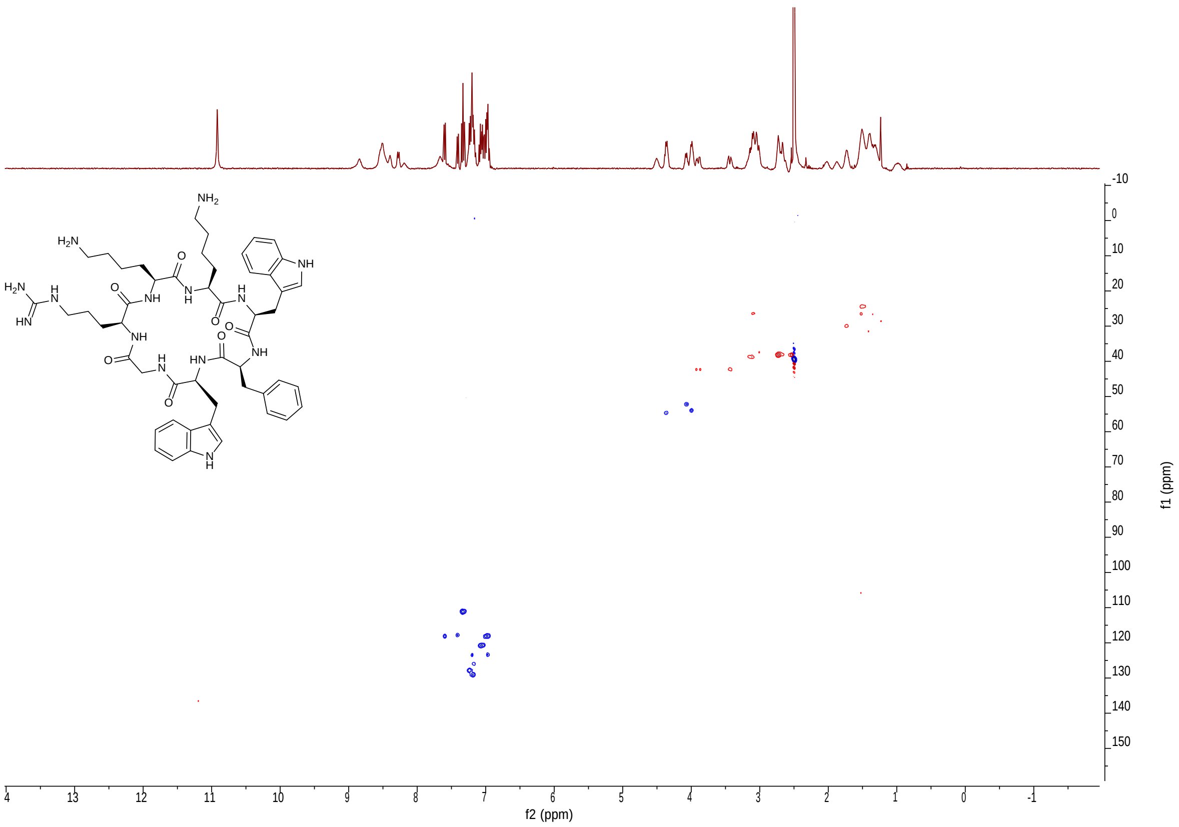

COSY NMR (DMSO-*d6*)

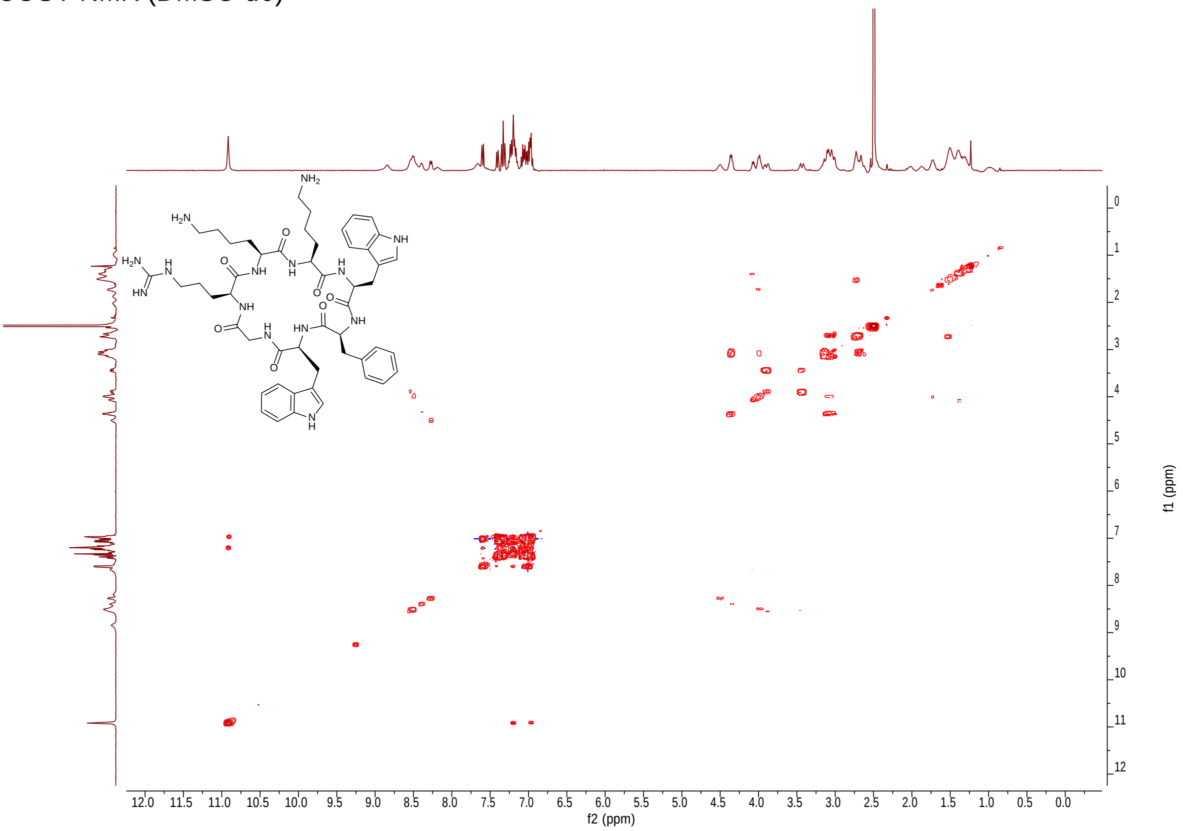

TOCSY NMR (DMSO-*d6*)

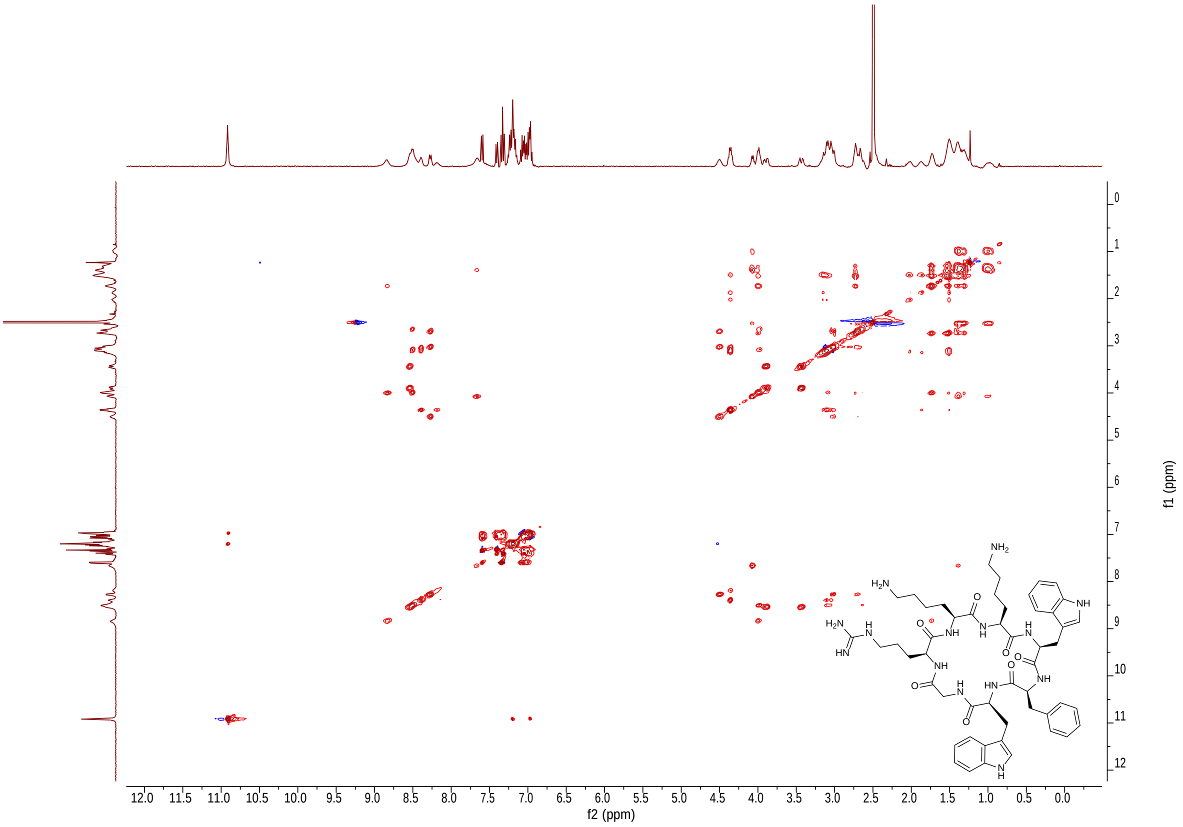

NOESY NMR (DMSO-*d6*)

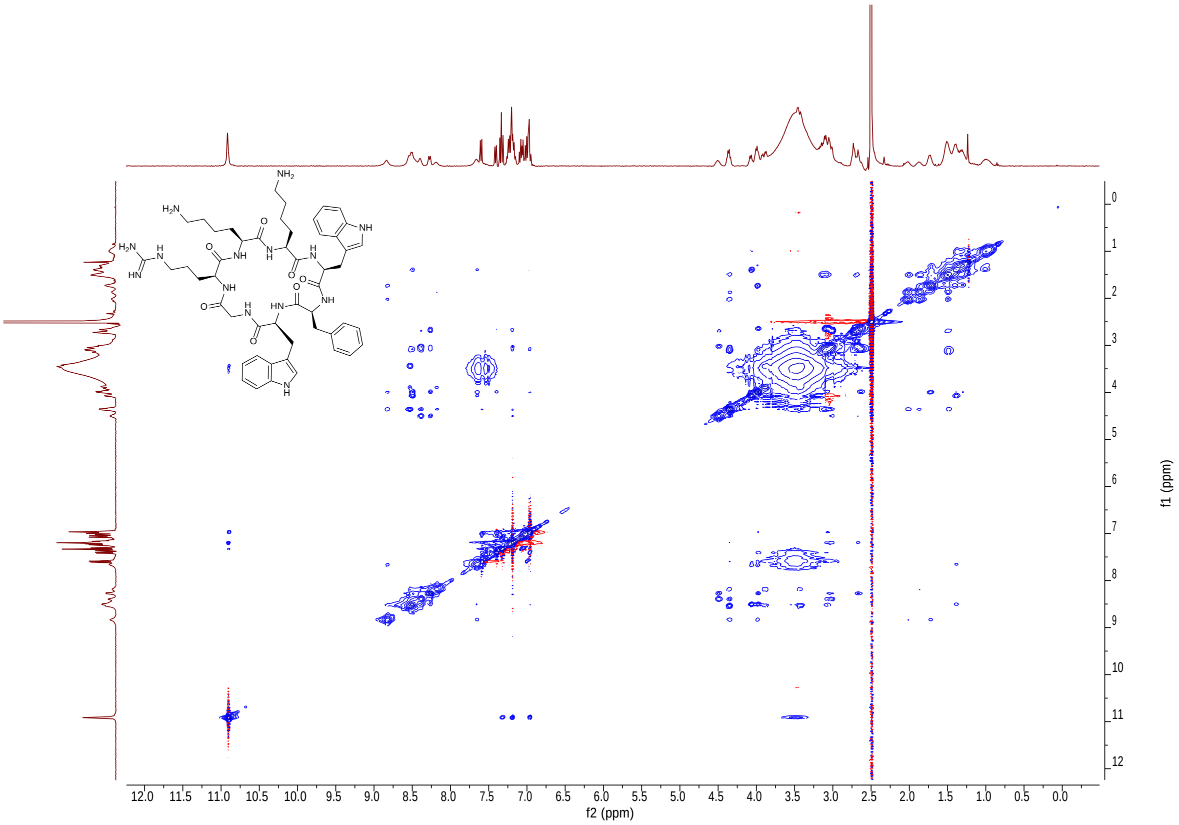

Supplementary Figure 25. NMR characterisation of cyclo(Arg-Lys-Lys-Trp-Phe-Trp-Gly) (**9**).

Supplementary Discussion

Considerations about the influence of the aryl iodide substitution pattern.

Initially, we explored the interaction of Fmoc-Trp-OH with the *p*-iodophenyl-BODIPY **1**[2] under similar conditions reported for this type of reactions[3] [5 mol% $Pd(OAc)_2$, **1** (4.0 eq.), $AgBF_4$ (1.0 eq.) and *o*-nitrobenzoic acid (1.5 eq.) in DMF under microwave irradiation at 150 ºC for 5 min]. Under these conditions, no reaction occurred. We then decided to test the *m*-iodophenyl-BODIPY **2** under the same reaction conditions and we observed the formation of the desired product (**3**). Next, we performed the reaction of Fmoc-Trp-OH with **2** applying an optimised arylation protocol[4] [5 mol% $Pd(OAc)_2$, **2** (1.5 eq.), $AgBF_4$ (1.0 eq.) and TFA (1.5 eq.) in DMF under microwave irradiation at 80 ºC for 20 min], which afforded the expected product **3** in 74% yield. The derivative **3** was recovered as a stable orange solid and properly characterised.

A plausible explanation lies in the nature of the arylation mechanism. Considering that the process is likely to involve a Pd(II)/Pd(IV) catalytic cycle, the C-H activation step may take place first to yield indole-Pd(II) species, where the oxidative addition to the Ar-I bond should occur, to be followed by the reductive elimination leading to the biaryl release and the regeneration of the catalyst.[5,6] As the intermediate Pd(II) species are quite electrophilic, it is reasonable to think that electron-rich aryl halides will insert faster than deactivated derivatives. Therefore, BODIPY groups, which are considered electron-withdrawing, should exert a stronger deactivating effect in para position and a milder one in meta position, thus facilitating the insertion of Pd(II) species into the C-I bond in the latter case.[7] Recent experimental results in similar reactions seem to support this hypothesis.[8]

Supplementary Methods

General experimental information.

Unless stated otherwise, all reactions were carried out under argon in dried glassware. Commercially available reactants were used without further purification. Thin-layer chromatography was conducted on Merck silica gel 60 F254 sheets and visualized by UV (254 nm and 365 nm). Silica gel (particle size 35–70 μm) was used for flash column chromatography. HPLC analysis were performed on an Agilent 1100 separations module connected to a multiwavelength UV detection system using Discovery or Symmetry columns (C_{18}, 5 μm, 4.6 × 150 mm), unless otherwise stated. NMR spectra were recorded on 400 or 500 MHz spectrometers. Chemical shifts (δ) are reported in ppm. Multiplicities are referred by the following abbreviations: s = singlet, d = doublet, t = triplet, dd = double doublet, dt = double triplet and m = multiplet. HRMS (ESI positive) were obtained with a LTQ-FT Ultra (Thermo Scientific) mass spectrometer. MALDI analysis was performed on a Bruker Ultraflex mass spectrometer. All microwave reactions were carried out in 10 mL sealed glass tubes in a focused mono-mode microwave oven ("Discover" by CEM Corporation) featured with a surface sensor for internal temperature determination. Cooling was provided by compressed air ventilating the microwave chamber during the reaction. When stated, the final crude was purified via flash column chromatography CombiFlash ISCO RF provided with dual UV detection.

Experimental procedures and characterisation data.

4,4-Difluoro-8-(4-iodophenyl)-1,3,5,7-tetramethyl-4-bora-*3a,4a*-diaza-*s*-indacene (1).

4-iodobenzaldehyde (500 mg, 2.2 mmol) was dissolved in anhydride DCM (50 mL) under N_2. Then, 2,4-dimethylpyrrole (492 µL, 4.8 mmol) and 2 drops of TFA were added and the reaction was stirred overnight at r.t in N_2 atmosphere or until the consumption of the aldehyde was complete (TLC). DDQ (490 mg, 2.2 mmol) dissolved in DCM (20 mL) was added dropwise (10-15 min) to the reaction mixture and the reaction was stirred for 15 min at r.t. Finally, TEA (4 mL, 45 mmol) and BF_3OEt_2 (4 mL, 30 mmol) were added and the mixture stirred for 3 h. Workup was done by diluting with DCM (50 mL) and washing with H_2O (4 x 100 mL). The organic layers were combined, dried over sodium sulphate, filtered and concentrated under vacuum. The crude was purified via flash column chromatography using and DCM/hexane gradient on silica gel. The expected compound was isolated as a red amorphous solid (290 mg, 29%).

Characterisation data: ^1H NMR (500 MHz, $CDCl_3$): δ 7.87 (d, J = 8.5 Hz, 2H), 7.08 (d, J = 8.5 Hz, 2H), 6.02 (s, 2H), 2.58 (s, 6H), 1.45 (s, 6H); ^{13}C NMR (125 MHz, $CDCl_3$): δ 155.9, 142.9, 138.3, 134.6, 131.1, 130.0, 121.5, 94.7, 14.7, 14.6; HRMS (m/z): $[M+H]^+$ calcd. for $C_{19}H_{18}BF_2IN_2$, 451.0654; found, 451.0651.

4,4-Difluoro-8-(3-iodophenyl)-1,3,5,7-tetramethyl -4-bora-*3a,4a*-diaza-*s*-indacene (2).

3-iodobenzaldehyde (500 mg, 2.2 mmol) was dissolved in anhydride DCM (50 mL) under N_2. Then, 2,4-dimethylpyrrole (492 µL, 4.8 mmol) and three drops of TFA were added and the reaction was stirred overnight at r.t in N_2 atmosphere or until the consumption of the aldehyde was complete (TLC). DDQ (490 mg, 2.2 mmol) dissolved in DCM (20 mL) was added dropwise (10-15 min) to the reaction mixture and the reaction was stirred for 15 min at r.t. Finally, TEA (4 mL, 45 mmol) and BF_3OEt_2 (4 mL, 30 mmol) were added and the mixture stirred for 3 h. Workup was done by diluting with DCM (50 mL) and washing with H_2O (4 x 100 mL). The organic layers were combined, dried over sodium sulfate, filtered and concentrated under vacuum. The crude was purified via flash column chromatography using and DCM/hexane gradient on silica gel. The expected compound was isolated as a red amorphous solid (401 mg, 41%).

Characterisation data: ^1H NMR (400 MHz, $CDCl_3$): δ 7.76 (dt, J = 7.7, 1.5 Hz, 1H), 7.62 (t, J = 1.6 Hz, 1H), 7.24 – 7.20 (m, 1H), 7.19 – 7.14 (m, 1H), 5.92 (s, 2H), 2.48 (d, J = 1.3 Hz, 6H), 1.36 (s, 6H); ^{13}C NMR (100 MHz, $CDCl_3$): δ 155.9, 142.9, 139.3, 138.0, 137.1, 136.8, 130.7, 127.3, 121.5, 94.3, 14.7, 14.6 (one quaternary carbon signal not seen); HRMS (m/z): $[M+H]^+$ calcd. for $C_{19}H_{18}BF_2IN_2$, 451.0654; found, 451.0651.

Fmoc-Trp(C_2-BODIPY)-OH (3).

Fmoc-Trp-OH (100 mg, 0.234 mmol), **2** (1.5 eq., 158 mg, 0.352 mmol), $AgBF_4$ (1.0 eq., 46 mg, 0.234 mmol), TFA (1.0 eq., 18 µL, 0.234 mmol) and $Pd(OAc)_2$ (0.05 eq., 2.6 mg, 0.0117 mmol) were placed in a microwave reactor vessel in 1.8 mL DMF. The mixture was heated under microwave irradiation (250 W) at 80°C for 20 min. EtOAc was added and the resulting suspension was filtered through Celite and concentrated under vacuum. The resulting crude was purified by flash column chromatography using and EtOAc/hexane gradient on silica gel. The expected adduct was isolated as a red solid (130 mg, 74%).

Characterisation data: ^1H NMR (400 MHz, $CDCl_3$): δ 8.12 (s, 1H), 7.69 – 7.56 (m, 4H), 7.48 (t, J = 7.7 Hz, 1H), 7.41 (t, J = 1.7 Hz, 1H), 7.37 (d, J = 4.9 Hz, 2H), 7.30 (t, J = 8.0 Hz, 3H), 7.25 – 7.21 (m, 1H), 7.20 – 7.13 (m, 3H), 7.07 (ddd, J = 8.0, 7.0, 1.1 Hz, 1H), 5.90 (s, 1H), 5.87 (s, 1H), 5.09 (d, J = 8.0 Hz, 1H), 4.55 (d, J = 7.5 Hz, 1H), 4.17 (q, J = 10.3, 9.4 Hz, 2H), 4.01 (s, 1H), 3.44 – 3.37 (m, 1H), 3.37 – 3.28 (m, 1H), 2.47 (s, 3H), 2.46 (s, 3H), 1.38 (s, 3H), 1.35 (s, 3H); ^{13}C NMR (100 MHz, $CDCl_3$): δ 174.8, 163.1, 156.0, 155.9, 143.9, 143.2, 141.4, 140.7, 136.1, 136.0, 135.1, 133.9, 131.5, 130.1, 129.1, 128.8, 127.9, 127.8, 127.2, 125.2, 123.2, 121.6, 120.5, 120.1, 119.3, 111.2, 108.2, 67.2, 47.2, 36.9, 28.0, 14.8, 14.7; HRMS (m/z): $[M+Na]^+$ calcd. for $C_{45}H_{39}BF_2N_4O_4$, 771.2930; found, 771.2925.

General procedures for SPPS. All peptides were manually synthesized in polystyrene syringes fitted with a polyethylene porous disc using Fmoc-based SPPS. Solvents and soluble reagents were removed by suction. The Fmoc group was removed with piperidine-DMF (1:4) (1 × 1 min, 2 × 5 min). Peptide synthesis transformations and washings were performed at r.t.

Resin loading (only for 2-chlorotrityl polystyrene resin). Fmoc-AA-OH (1 eq.) was attached to the resin (1 eq.) with DIPEA (3 eq.) in DCM at r.t for 10 min and then DIPEA (7.0 eq.) for 40 min. The remaining trityl groups were capped adding 0.8 µL MeOH/mg resin for 10 minutes. After that, the resin was filtered and washed with DCM (4 x 1 min), DMF (4 x 1 min). The loading of the resin was determined by titration of the Fmoc group. Peptide elongation. After the Fmoc group was removed, the resin was washed with DMF (4 x 1 min), DCM (3 x 1 min), DMF (4 x 1 min). Unless otherwise noted, standard coupling procedures used DIC (3 eq.) and OxymaPure (3 eq.) in DMF for 1 h and 5-min of pre-activation. The completion of the coupling was monitored by the Kaiser test.[9] Then, the resin was filtered and washed with DCM (4 x 1 min) and DMF (4 x 1 min). Final cleavage (for Sieber amide and for 2-chlorotrityl polystyrene resins). The resin bound peptide was treated for 5 times with 1% TFA in DCM (1 min in each treatment) and washed with DCM. The combined filtered mixtures were poured over DCM and evaporated under vacuum. Then, the residue was dissolved in ACN:H_2O and liophilised.

General procedures for linear peptides 4-7. Syntheses were performed on Sieber amide resin (0.69 mmol/g). Amino acid **3** (1.5 eq.) was incorporated with a 5-min pre-activation using DIC (1.5 eq.) and OxymaPure (1.5 eq.) in DMF for 1h. For Fmoc-Arg-OH (3 eq.), we performed several treatments (up to 9) with DIC (3 eq.) and HOBt (3 eq.) in DMF for 15 min without pre-activation. The rest of amino acids (Fmoc-AA-OH, 3 eq.) were incorporated with a 5-min pre-activation with DIC (3 eq.) and OxymaPure (3 eq.) in DMF for 1 h. Peptides were purified by semi-preparative RP-HPLC (XBRIDGE™ BEH 130,

C_{18}, 5 µM OBD 19×50 mm column). Mobile phase: ACN (0.1% HCOOH)/H_2O (0.1% HCOOH); flow rate: 20 mL/min. Pure fractions were lyophilised furnishing the corresponding peptides.

H-Arg-Lys-Lys-Trp-Phe-Trp-NH_2 (4). Synthesised as previously reported.[10]

White powder (5.0 mg).

Characterisation data: HPLC: $t_{R:}$ 3.37 min (95% purity); HRMS (m/z): $[M+H]^+$ calcd. for $C_{49}H_{67}N_{13}O_7$, 950.5365; found, 950.5364.

H-Arg-Lys-Lys-Trp(C_2-BODIPY)-Phe-Trp-NH_2 (5).

Red powder (3.6 mg).

Characterisation data: HPLC: $t_{R:}$ 3.28 min (90% purity); HRMS (m/z): $[M+H]^+$ calcd. for $C_{68}H_{85}BF_2N_{16}O_6$,1271.6977; found, 1271.6947.

H-Ala-Ala-Ala-Trp(C_2-BODIPY)-Phe-Trp-NH_2 (5a).

Red powder (20 mg).

Characterisation data: HPLC: $t_{R:}$ 6.60 min (96% purity). HRMS (m/z): $[M+H]^+$ calcd. for $C_{59}H_{64}BF_2N_{11}O_6$: 1072.5175; found, 1072.5183.

H-Arg-Lys-Lys-Trp(C_2-BODIPY)-Ala-Ala-NH_2 (5b).

Red powder (7 mg).

Characterisation data: HPLC: $t_{R:}$ 4.77 min (99% purity). HRMS (m/z): $[M+H]^+$ calcd. for $C_{54}H_{76}BF_2N_{15}O_{6:}$ 1080.6237; found, 1080.6250.

H-Arg-Lys-Lys-Trp-Phe-Trp(C_2-BODIPY)-NH_2 (6).

Red powder (1.8 mg).

Characterisation data: HPLC: $t_{R:}$ 3.95 min (90% purity); HRMS (m/z): $[M+H]^+$ calcd. for $C_{68}H_{85}BF_2N_{16}O_6$, 1271.6977; found: 1271.6937.

H-Arg-Lys-Lys-Trp(C_2-BODIPY)-Phe-Trp(C_2-BODIPY)-NH_2 (7).

Red powder (16.8 mg).

Characterisation data: HPLC: $t_{R:}$ 6.24 min (91% purity); HRMS (m/z): $[M+H]^+$ calcd. for $C_{87}H_{103}B_2F_4N_{18}O_6$, 1593.8449; found, 1593.8532.

Cyclo(Arg-Lys-Lys-Trp(C_2-BODIPY)-Phe-Trp-Gly) (8). The synthesis was performed on 91 mg of 2-chlorotrityl polystyrene resin (0.94 mmol/g). Amino acid **3** (1.5 eq.) was incorporated with PyBOP (1.5 eq.), HOBt (1.5 eq.) and DIPEA (2.0 eq.) in DMF for 1 h. Other Fmoc amino acids (3 eq.) were incorporated with a 5 min pre-activation with DIC (3 eq.) and OxymaPure (3 eq.) in DMF for 1 h. After cleavage as described above, the protected linear peptide (119 mg, 0.073 mmol) was dissolved in 1.3 mL of DMF (0.055 M). DIPEA (2.5 eq., 32 µL, 0.182 mmol) and HATU (1.0 eq., 28 mg, 0.073 mmol) were added. The solution was stirred at r.t. until the cyclisation was complete (approx. 2 h). The cyclic peptide was precipitated by adding H_2O to the solution. The precipitate was washed with H_2O, decanted and dried, obtaining 109 mg of crude protected cyclic peptide (87% yield). The crude protected macrocycle (108 mg, 0.067 mmol) and 20% $Pd(OH)_2$-C (54 mg) were dissolved in 5% HCOOH/MeOH (10.8 mL), previously purged with Ar. Then, the reaction flask was flushed again with Ar, evacuated and filled with H_2. The reaction mixture was stirred under H_2 for 72 h (H_2 balloons were refilled periodically during the reaction along with re-addition of $Pd(OH)_2$-C (4 times). The catalyst was removed by filtration

and the filtrate was evaporated to afford 46 mg of the crude deprotected peptide. The final peptide was purified by PoraPak Rxn RP 60 cc reverse phase column. Mobile phase: ACN (0.1% HCOOH)/H_2O (0.1% HCOOH). Pure fractions were lyophilised furnishing the corresponding peptide.

Red powder (30 mg).

Characterisation data: ^{1}H NMR (600 MHz, CD_3OD): δ 8.55 (s, 3H), 7.89 – 7.84 (m, 1H), 7.71 (t, J = 7.7 Hz, 1H), 7.65 (d, J = 7.8 Hz, 1H), 7.60 – 7.55 (m, 2H), 7.42 (dt, J = 8.1, 0.9 Hz, 1H), 7.40 – 7.36 (m, 2H), 7.18 – 7.12 (m, 5H), 7.11 (s, 1H), 7.06 (m, 3H), 6.94 (m, 1H), 6.10 (s, 1H), 6.06 (s, 1H), 4.45 (t, J = 7.4 Hz, 1H), 4.32 (d, J = 10.5 Hz, 1H), 4.25 (m, 1H), 4.17 – 4.07 (m, 2H), 4.04 (d, J = 16.5 Hz, 1H), 4.00 (m, 1H), 3.63 (dd, J = 14.9, 9.5 Hz, 1H), 3.51 – 3.45 (m, 1H), 3.38 (m, 1H), 3.30 (1H), 3.27 – 3.23 (m, 2H), 3.15 (dd, J = 14.2, 7.5 Hz, 1H), 3.10 (dd, J = 14.0, 5.9 Hz, 1H), 2.90 (td, J = 8.6, 4.2 Hz, 2H), 2.72 (t, J = 7.7 Hz, 2H), 2.63 (dd, J = 14.2, 8.5 Hz, 1H), 2.51 (s, 3H), 2.50 (s, 3H), 2.09 – 1.88 (m, 4H), 1.76 (m, 1H), 1.67 (m, 3H), 1.52 (m, 7H), 1.48 – 1.41 (m, 4H), 1.37 (m, 2H), 1.26 – 1.14 (m, 2H); HPLC: $t_{R:}$ 4.45 min (98% purity); HRMS (m/z): $[M+H]^+$ calcd. for $C_{70}H_{86}BF_2N_{16}O_7$, 1311.6926; found, 1311.6864.

Cyclo(Arg-Lys-Lys-Trp-Phe-Trp-Gly) (9). The synthesis was performed on 500 mg of 2-chlorotrityl polystyrene resin (1.0 mmol/g). Fmoc amino acids (3 eq.) were incorporated with a 5-min pre-activation with DIC (3 eq.) and OxymaPure (3 eq.) in DMF for 1 h. After cleavage as described above, the fully protected linear peptide (300 mg, 0.18 mmol) was dissolved in 3.5 mL of DMF (0.052 M). DIPEA (2.5 eq., 79 µL, 0.18 mmol) and HATU (1.0 eq., 69 mg, 0.18 mmol) were added. The solution was stirred at r.t until the cyclisation was complete (2 h). The cyclic peptide was precipitated by adding H_2O to the solution and the precipitate was washed with H_2O, decanted and dried. The protected linear peptide was dissolved in TFA:TIS:H_2O (95:2.5:2.5) and the solution was stirred at r.t. for 2 h. The resulting solution was evaporated under vacuum, washed with Et_2O, dissolved in ACN:H_2O and liophilised to afford 231 mg of the crude peptide (72% yield). 145 mg of peptide were purified by PoraPak Rxn RP 60 cc reverse phase column. Mobile phase: ACN (0.1% HCOOH)/H_2O (0.1% HCOOH). Pure fractions were lyophilised furnishing the corresponding pure cyclic peptide.

White powder (51 mg).

Characterisation data: ^{1}H NMR (600 MHz, DMSO-d_6): δ 10.92 (s, 2H), 8.92 – 8.78 (m, 1H), 8.59 – 8.45 (m, 3H), 8.44 – 8.35 (m, 1H), 8.27 (d, J = 8.4 Hz, 1H), 8.23 – 8.13 (m, 1H), 7.73 – 7.63 (m, 1H), 7.60 (d, J = 7.9 Hz, 1H), 7.41 (d, J = 7.9 Hz, 1H), 7.38 – 7.29 (m, 1H), 7.26 – 7.13 (m, 7H), 7.11 – 7.04 (m, 2H), 7.02 (dd, J = 3.9, 1.1 Hz, 1H), 7.01 – 6.94 (m, 3H), 4.56 – 4.44 (m, 1H), 4.36 (q, *J* = 6.9 Hz, 2H), 4.14 – 4.04 (m, 1H), 4.00 (q, J = 6.7 Hz, 2H), 3.90 (dd, J = 16.9, 6.0 Hz, 1H), 3.50 – 3.38 (m, 1H), 3.21 – 2.97 (m, 7H), 2.80 – 2.62 (m, 4H), 2.10 – 1.97 (m, 1H), 1.95 – 1.81 (m, 1H), 1.81 – 1.67 (m, 2H), 1.58 – 1.27 (m, 12H), 1.06 – 0.91 (m, 1H); HPLC: $t_{R:}$ 1.55 min (98% purity); HRMS (m/z): $[M]^+$ calcd. for $C_{51}H_{68}N_{14}O_7$, 988.5395; found, 988.5387.

Supplementary References

[1] Seybold, P. G., Gouterman, M. & Callis, J. Calorimetric, photometric and lifetime determinations of fluorescence yields of fluorescein dyes. Photochem. Photobiol. **9**, 229-242 (1969).

[2] Tahtaoui, C. et al. Convenient Method To Access New 4,4-Dialkoxy- and 4,4-Diaryloxy-diaza-s-indacene Dyes: Synthesis and Spectroscopic Evaluation. J. Org. Chem. **72**, 269–272 (2007).

[3] Ruiz-Rodriguez, J., Albericio, F. & Lavilla, R. Postsynthetic Modification of Peptides: Chemoselective C-Arylation of Tryptophan Residues. Chem. Eur. J. **16**, 1124–1127 (2010).

[4] Preciado, S., Mendive-Tapia, L., Albericio F. & Lavilla, R. Synthesis of C-2 Arylated Tryptophan Amino Acids and Related Compounds through Palladium-Catalyzed C–H Activation. J. Org. Chem. **78**, 8129–8135 (2013).

[5] Ackermann, L. Carboxylate-Assisted Transition-Metal-Catalyzed C−H Bond Functionalizations: Mechanism and Scope. Chem. Rev. **111**, 1315–1345 (2011).

[6] Chiong, H. A., Pham Q. & Daugulis, O. Two Methods for Direct ortho-Arylation of Benzoic Acids. J. Am. Chem. Soc. **129**, 9879–9884 (2007).

[7] Yu, J. Q. & Shi, Z. C-H Activation. Topics in Current Chemistry, Springer, Berlin Heidelberg (2010).

[8] Feng, J., Lu, G., Lv, M. & Cai, C. Palladium catalyzed direct C-2 arylation of indoles. J. Organomet. Chem. **761**, 28–31 (2014).

[9] Kaiser, E., Colescott, R. L., Bossinger C. D. & Cook, P. I. Color test for detection of free terminal amino groups in the solid-phase synthesis of peptides. Anal. Biochem. **34**, 595-598 (1970).

[10] Lopez-Garcia, B., Perez-Paya, E. & Marcos, J. F. Identification of novel hexapeptides bioactive against phytopathogenic fungi through screening of a synthetic peptide combinatorial library. Appl. Environ. Microbiol. **68**, 2453-2460 (2002).
